# Urban–rural disparities in HPV prevalence and genotype distribution: a large-scale cervical cancer screening study in Xi’an, China

**DOI:** 10.1186/s12879-026-13647-2

**Published:** 2026-05-28

**Authors:** Jingrui Wu, Jianhua Yi, Le Fang, Shuya Lei, Haihui Yang, Mangni Ma, Yongle Hao, Hongrae Kim, Hyungsoo Shin, Wansuk Choi

**Affiliations:** 1 College of Medical Technology, Xi’an Medical College, Xi’an, Shaanxi China; 2 Department of Clinical Laboratory, Affiliated Hospital of Xi’an Medical College, Xi’an, Shaanxi China; 3 Department of Clinical Laboratory, Norinco General Hospital, Xi’an, Shaanxi China; 4 Department of Basic Sciences, Xi’an Medical College, Xi’an, Shaanxi China; 5 Department of Physical Therapy, Kyungwoon University, Gumi, Gyeongsangbuk-do, Republic of Korea; 6 Department of Public Health, Kyungwoon University, Gumi, Gyeongsangbuk-do Republic of Korea

**Keywords:** Human papillomavirus, Cervical cancer screening, HPV genotype, Urban–rural disparity, Healthcare equity, China, HPV52, Co-infection, Follow-up adherence

## Abstract

**Background:**

Cervical cancer remains a major public health challenge in China, with persistent urban–rural disparities in screening coverage. The epidemiological landscape of HPV genotype distribution and screening outcomes across geographic settings remains incompletely characterized. We compared HPV prevalence, genotype distribution, and screening outcomes between urban and rural populations in Xi’an, China, during the post-COVID-19 pandemic period (2023–2025).

**Methods:**

This retrospective cross-sectional study analysed HPV testing records from 16,365 women (11,449 urban, 4,916 rural) screened between January 2023 and December 2025. Primary urban–rural comparisons were restricted to the 15 high-risk genotypes common to both detection platforms (HPV16, 18, 31, 33, 35, 39, 45, 51, 52, 53, 56, 58, 59, 66, 68). Histopathological follow-up was characterised within the rural sub-cohort only (matched urban cascade data unavailable). Statistical analyses included chi-square with Bonferroni correction, Wilson 95% CIs, and network analysis for co-infection patterns; significance set at *p* < 0.05.

**Results:**

Age-standardised HPV prevalence was higher in urban (14.3%, 95% CI: 13.6–15.0%) than rural populations (12.4%, 95% CI: 11.4–13.4%; *p* = 0.007). HPV52 predominated in both settings (urban: 3.1%, rural: 3.2%), followed by HPV16 (2.4% in both) and HPV58 (urban: 2.4%, rural: 1.6%). Significant urban–rural differences: HPV58 (urban higher; *p* = 0.002), HPV33 (rural higher; *p* = 0.003), HPV39 (rural higher; *p* = 0.012); HPV16, HPV18, HPV52 did not differ. Urban women showed a U-shaped age distribution, while rural women showed a gradual age-related increase. Multiple infections were similar (urban: 4.0%, rural: 3.4%; *p* = 0.087). HPV52–HPV53 was the most frequent co-infection pair in both networks (urban: 32; rural: 16). Histopathological follow-up in the rural sub-cohort was 11.5% (70/609 HPV-positive women).

**Conclusions:**

Urban–rural differences in HPV prevalence and genotype distribution were observed in Northwest China. The crude urban excess (15.5% vs. 12.4%) was substantially attenuated but not eliminated after platform-comparable, age-standardised analysis (14.3% vs. 12.4%; *p* = 0.007), reflecting detection-platform asymmetry, age structure, and differential healthcare access. HPV52 predominance underscores the need for region-specific vaccination strategies beyond HPV16/18. The 11.5% rural histopathological follow-up rate, far below the WHO 70% minimum, highlights a critical cascade-of-care gap; prospective matched-cohort studies are required to characterise the urban cascade and guide equitable diagnostic strengthening.

**Supplementary Information:**

The online version contains supplementary material available at 10.1186/s12879-026-13647-2.

## Introduction

Cervical cancer remains a major global health burden, with an estimated 604,000 new cases and 342,000 deaths in 2020, making it the fourth most common cancer among women worldwide [1]. The disease disproportionately affects low- and middle-income countries (LMICs), which account for approximately 90% of cervical cancer deaths [2]. In China, cervical cancer represents a significant public health challenge, with an estimated 109,700 new cases and 59,000 deaths in 2020, accounting for approximately 18% of the global disease burden [3]. Unlike high-income countries where organized screening programs have led to substantial declines in incidence and mortality, China has experienced concerning upward trends, with age-standardized incidence rates increasing from 9.10 per 100,000 women in 2005 to 18.10 per 100,000 in 2018 [4]. Rural areas demonstrate particularly alarming trends, with higher annual percentage changes in both incidence and mortality compared to urban regions, reflecting persistent healthcare disparities [5].

Persistent infection with high-risk human papillomavirus (HR-HPV) is the necessary and primary cause of virtually all cervical cancers, with HPV DNA detected in 99.7% of cervical cancer specimens worldwide [6, 7]. The natural history of HPV infection is characterized by rapid clearance in most cases, with approximately 70% of infections clearing within one year and 90% within two years [8]. However, persistent infections, particularly with high-risk genotypes, can progress to cervical intraepithelial neoplasia (CIN), with a subset advancing to invasive cancer over decades [9]. Low-grade squamous intraepithelial lesions frequently regress spontaneously, particularly in younger women, emphasizing the importance of distinguishing transient infections from clinically significant persistent infections [10]. Among the human papillomavirus genotypes classified as carcinogenic by the International Agency for Research on Cancer (IARC) [11–13] (12 Group 1 types: HPV16, 18, 31, 33, 35, 39, 45, 51, 52, 56, 58, 59; plus HPV68, Group 2A “probably carcinogenic”; HPV53 and HPV66 are classified as Group 2B “possibly carcinogenic”), HPV16 and HPV18 are globally the most prevalent, accounting for approximately 70% of cervical cancer cases [14, 15]. However, substantial geographic and ethnic variations exist in HPV genotype distribution, with important implications for screening strategies and vaccine effectiveness [16, 17]. In Chinese populations, the epidemiological landscape differs markedly from Western countries, with HPV52 and HPV58 demonstrating notably higher prevalence than in other regions [18, 19]. Recent large-scale meta-analyses have identified HPV16, HPV52, and HPV58 as the three most common genotypes in Chinese women with cervical intraepithelial neoplasia (CIN) and invasive cervical cancer, with HPV52 prevalence rates approaching or exceeding those of HPV16 in certain regions [20, 21]. This unique genotype distribution pattern has critical implications for vaccine development, as current bivalent and quadrivalent vaccines targeting HPV16/18 may provide suboptimal protection for the Chinese population. The nine-valent vaccine, which includes HPV31, 33, 45, 52, and 58, theoretically offers broader coverage against the genotypes most prevalent in Chinese women; however, its widespread deployment in China has lagged substantially behind other countries [22, 23]. This delay is partly attributable to the later regulatory approval timeline in China. Although approved by the US FDA in December 2014, the nine-valent vaccine was not approved by China’s National Medical Products Administration (NMPA) until April 2018. Furthermore, its widespread deployment has been constrained by significant supply–demand imbalances and initial age eligibility restrictions (originally limited to females aged 16–26 years, subsequently expanded to 9–45 years in 2022) [22, 23].

International consensus guidelines recommend organized, population-based cervical cancer screening programs as the cornerstone of prevention [24]. The World Health Organization (WHO) has endorsed HPV testing as the preferred primary screening method, with higher sensitivity for detecting high-grade cervical intraepithelial neoplasia compared to cytology-based approaches [25]. Multiple comparative screening studies, including randomized controlled trials and large multicentre evaluations, have demonstrated that HPV-based screening reduces cervical cancer incidence and mortality more effectively than cytology alone, supporting its adoption in national guidelines globally [26–29]. HPV DNA testing demonstrates superior performance characteristics for primary screening, with pooled analyses showing significantly higher sensitivity for detecting CIN2+ lesions compared to cytology [30]. Advanced screening technologies including HPV mRNA assays and deep learning-based automated cytology interpretation show promise for improving screening accuracy and efficiency [31, 32]. HPV genotyping provides valuable triage information for women with positive primary screening results, helping stratify risk and guide clinical management decisions [33]. The integration of self-sampling methods has emerged as a strategy to improve screening coverage, with meta-analyses demonstrating comparable accuracy between self-collected and clinician-collected samples when using validated HPV tests [34]. The optimal screening strategy, however, varies by resource setting, healthcare infrastructure, and population characteristics [35]. In China, multiple screening modalities coexist, including visual inspection with acetic acid (VIA), cytology, and HPV DNA testing, reflecting diverse implementation contexts across regions with different economic development levels [36, 37]. The National Health Commission of China has gradually shifted toward HPV-based screening in recent years, aligning with international evidence-based recommendations [35]. Long-term follow-up studies have demonstrated that several consecutive negative HPV and cytology co-testing results provide substantial reassurance against future cervical cancer risk, supporting extended screening intervals for low-risk women [38].

China launched the National Cervical Cancer Screening Program in Rural Areas (NACCSPRA) in 2009, initially targeting rural women aged 35–64 years with free screening services [39]. The program has progressively expanded its geographic coverage and gradually extended to urban areas, representing one of the world’s largest population-based cervical cancer screening initiatives. Screening coverage has improved substantially, increasing from 36.8% in 2018–2019 to 51.5% in 2023–2024 among the target population [40]. However, significant urban–rural disparities persist, with rural screening coverage (48.2%) remaining below urban levels (54.1%) and falling short of the national target of 80% coverage by 2030 outlined in the Healthy China 2030 initiative [41]. These disparities reflect broader structural inequalities in healthcare infrastructure, personnel capacity, quality of services, and health literacy between urban and rural regions [42]. Rural healthcare facilities often face challenges including inadequate equipment, insufficiently trained personnel for colposcopy and pathology services, and limited capacity for timely follow-up of abnormal results [43, 44]. Innovative approaches including HPV self-sampling and digital colposcopy have shown feasibility for expanding screening access in resource-limited rural settings [45].

While screening coverage represents an important metric, the ultimate effectiveness of cervical cancer prevention programs depends critically on the complete continuum of care from initial screening through diagnosis and treatment. International guidelines emphasize that screening programs must ensure not only high participation rates but also adequate follow-up of positive screening results, timely colposcopy examination, histopathological confirmation, and appropriate management of precancerous lesions [37]. Unfortunately, substantial gaps exist in this clinical pathway across many settings. Studies from both high-income and LMICs have documented concerning rates of loss to follow-up at multiple steps, with follow-up completion rates often below 50% among women with abnormal screening results [45]. In China, systematic data on follow-up adherence remain limited, but available evidence suggests significant challenges. Studies have reported wide variation in colposcopy completion rates (ranging from 30% to 70%) and treatment initiation rates for confirmed CIN lesions [46]. Patient-level barriers include limited awareness of the clinical significance of abnormal results, financial constraints, geographic distance to specialized facilities, and cultural factors. System-level barriers encompass inadequate referral mechanisms, insufficient communication between screening and diagnostic facilities, lack of patient tracking systems, and shortage of colposcopy and pathology services particularly in rural areas [47].

Despite the substantial body of literature on cervical cancer screening in China, several critical knowledge gaps remain. First, most existing studies have examined either urban or rural populations separately, with limited direct comparative analyses of HPV prevalence, genotype distribution, and screening outcomes across both settings simultaneously using standardized methodologies [46]. Such comparative data are essential for understanding the extent of health disparities and designing targeted interventions. Second, while numerous studies have reported HPV prevalence rates, many lack detailed stratification by disease severity, age groups, and temporal trends, limiting insights into risk patterns and vulnerable populations [20]. Third, comprehensive analyses of clinical management pathways following positive screening results are scarce, particularly regarding urban–rural differences in colposcopy completion rates, histopathological follow-up, and factors associated with loss to follow-up. Fourth, HPV genotype distribution data specific to the post-COVID-19 pandemic period (2023–2025) are limited, despite potential impacts of pandemic-related healthcare disruptions on screening behaviors, healthcare-seeking patterns, and HPV epidemiology [48]. Finally, most published studies have utilized relatively small sample sizes or focused on specific geographic regions, potentially limiting generalizability and statistical power to detect important subgroup differences.

The present study addresses these knowledge gaps through a large-scale comparative analysis of HPV infection patterns, genotype distribution, and cervical cancer screening outcomes between urban and rural hospital settings in Xi’an, Shaanxi Province, China. Using data from 16,365 women (11,449 urban and 4,916 rural) screened between January 2023 and December 2025, we systematically compared: (1) overall HPV prevalence and temporal trends; (2) distribution of 25 HPV genotypes with particular focus on high-risk types according to IARC classification; (3) patterns of multiple HPV co-infections and specific genotype combinations; (4) age-specific prevalence patterns and peak infection periods; and (5) histopathological follow-up outcomes within the rural sub-cohort, for which confirmed-pathology records were available (matched urban cascade-of-care data were not available for the present analysis). By characterising HPV epidemiology in this Northwest China population—and, within the rural sub-cohort, the screening-to-diagnosis pathway—using standardised 25-type HPV genotyping, this study provides critical insights into healthcare delivery gaps and identifies specific intervention points to strengthen cervical cancer prevention programs. Our findings inform resource allocation, region-specific screening strategies, vaccine policy aligned with China’s genotype distribution, and healthcare-equity initiatives aimed at reducing cervical cancer burden.

## Methods

### Study design and setting

This retrospective, comparative cross-sectional study was conducted in Xi’an, Shaanxi Province, Northwest China, from January 2023 to December 2025. Urban participants (*n* = 11,449) were recruited from tertiary hospitals and community health centers in metropolitan Xi’an. Rural participants (*n* = 4,916) were recruited from township health centers and village clinics in counties surrounding Xi’an through government-sponsored cervical cancer screening programs.

### Study population

Eligible records were those of female individuals aged 15–100 years with a valid HPV genotyping result generated during the study period. For individuals with multiple HPV testing episodes, only one record per unique individual was retained on the basis of personal identifiers; remaining duplicate testing records were excluded prior to analysis. Records corresponding to male individuals, records with missing age data, or records with age outside the predefined range of 15–100 years were also excluded; the latter two exclusion categories were applied jointly via numeric range filtering on the age field, since records with non-numeric or blank age values could not be assigned to an age stratum. As all analysed specimens were cervical exfoliated-cell samples obtained by endocervical brush sampling, presence of a cervix at the time of screening was an inherent operational prerequisite for sampling, and hysterectomy history was routinely ascertained by the attending clinician during pre-sampling consultation in standard screening practice; hysterectomy status was, however, not separately captured as a structured field in the source records and was therefore not verifiable retrospectively at the record level. Because this study was a retrospective analysis of routinely collected hospital screening records, individual-level information on potentially relevant clinical exclusion conditions—including previous hysterectomy, current pregnancy, menstruation at the time of sampling, prior cervical cancer, and recent cervical procedures—was not consistently available in the source datasets and could not be applied at the record level; this is acknowledged as a limitation of the present analysis (see Limitations).

### Sample collection and HPV detection

Cervical specimens were collected by trained healthcare providers using a cervical brush rotated 360° three times in the cervical canal. Samples were immediately placed in preservation medium and transported to the laboratory within 4 hours. DNA was extracted using a commercial kit (Hunan Sansure Biotech, Changsha, China) according to the manufacturer’s protocol; DNA quality was subsequently assessed by a PCR fluorescent-probe assay (Hunan Sansure Biotech), and samples meeting both quality criteria—sample DNA Ct ≤ 39 and internal control Ct ≤ 40—were considered suitable for downstream PCR amplification.

HPV genotyping was performed using the Human Papillomavirus Nucleic Acid Genotyping Detection Kit (PCR-Fluorescent Probe Method; Hunan Sansure Biotech, Changsha, China), which simultaneously detects 25 HPV types as labelled by the manufacturer: 15 types labelled high-risk (HPV16, 18, 31, 33, 35, 39, 45, 51, 52, 53, 56, 58, 59, 66, 68; comprising the 13 IARC Group 1/2A genotypes plus HPV53 and HPV66, which are classified by IARC as Group 2B “possibly carcinogenic” [11, 12] but are grouped with high-risk genotypes by Chinese commercial-assay convention), three labelled probable high-risk (HPV26, 73, 82; all IARC Group 2B), and seven labelled low-risk (HPV6, 11, 42, 43, 70, 81, 83; HPV70 is classified by IARC as Group 2B but is included in the low-risk panel by the manufacturer). PCR amplification was performed with the following conditions: initial denaturation at 50 °C for 2 min and 94 °C for 5 min, followed by 40 cycles of 94 °C for 15 s and 57 °C for 30 s, with a final extension at 72 °C for 10 min.

Quality control measures included positive and negative controls in each PCR run, repeat testing of 5% randomly selected samples (concordance rate > 99%), and quarterly inter-laboratory quality assurance. External quality assessment was conducted annually through Shaanxi Provincial Clinical Laboratory Center proficiency testing programs with 100% pass rate throughout the study period.

Two HPV detection platforms operated during the study period. Urban specimens were tested either with a 15-type high-risk-only assay (covering HPV16, 18, 31, 33, 35, 39, 45, 51, 52, 53, 56, 58, 59, 66, and 68) or with the comprehensive 25-type assay, which is a strict superset of the 15-type panel and additionally detects three probable high-risk types (HPV26, 73, 82) and seven low-risk types (HPV6, 11, 42, 43, 70, 81, 83). All rural specimens were tested with the 15-type high-risk-only assay. To minimise platform-induced detection bias, the primary urban–rural comparisons reported in this study were restricted to the 15 high-risk genotypes covered by both assays (Table 3); for the 10 additional genotypes detected only by the 25-type panel, the corresponding rural counts of zero in Table 3 reflect non-testing rather than confirmed absence of infection, and these comparisons are reported for descriptive completeness only.

### Statistical analysis

Data were entered into Microsoft Excel 2019 (Microsoft Corporation, Redmond, WA, USA) with double-entry verification to ensure accuracy. Statistical analyses were performed using Python 3.9 with pandas (v1.5.3), NumPy (v1.24.2), SciPy (v1.10.1), statsmodels (v0.14.0), matplotlib (v3.7.1), seaborn (v0.12.2), and networkx (v3.1). Data normality was assessed using the Shapiro-Wilk test. Continuous variables were presented as mean ± standard deviation (SD) for normally distributed data or median with interquartile range (IQR) for non-normally distributed data. Categorical variables were presented as frequencies and percentages.

HPV positivity rates were calculated with 95% confidence intervals (CI) using the Wilson score method to account for proportions near boundaries. Urban and rural populations were compared using independent samples t-test for normally distributed continuous variables or Mann-Whitney U test for non-normally distributed continuous variables. For categorical variables, chi-square test was used when expected frequencies were ≥ 5 in all cells; otherwise, Fisher’s exact test was employed. Statistical comparisons in Table 1 are restricted to demographic characteristics (age and recruitment setting); HPV infection metrics represent the primary study outcomes and are compared between groups using multivariable models with appropriate covariate adjustment, as reported in Tables 2–6.

**Table 1:** Baseline characteristics and HPV prevalence by geographic setting

Characteristic	Urban	Rural	p-value
Sample size, n	11,449	4,916	-
Age, mean ± SD (years)	42.8 ± 10.4	47.0 ± 10.3	<0.001
Age groups, n (%)	—	—	—
<30	917 (8.0%)	139 (2.8%)	—
30–39	3,971 (34.7%)	1,239 (25.2%)	—
40–49	3,532 (30.8%)	1,326 (27.0%)	—
50–59	2,237 (19.5%)	1,614 (32.8%)	—
≥60	792 (6.9%)	598 (12.2%)	—
HPV Positive, n (%)	1,780 (15.55%)	609 (12.39%)	<0.001
High-Risk HPV, n (%)	1,391 (12.15%)	542 (11.03%)	0.044
Infection Type, n (%)	—	—	—
Negative	9,669 (84.5%)	4,307 (87.6%)	—
Single	1,317 (11.5%)	444 (9.0%)	—
Multiple	463 (4.0%)	165 (3.4%)	—

Baseline characteristics and HPV infection patterns by geographic setting (urban: *n* = 11,449; rural: *n* = 4,916). Statistical comparisons restricted to demographic characteristics (age and recruitment setting); HPV infection metrics constitute primary study outcomes with confounder-adjusted comparisons in Tables 2–6. CI, confidence interval; HPV, human papillomavirus; HR-HPV, high-risk HPV—defined here as the 13 IARC group 1/2A genotypes (HPV16, 18, 31, 33, 35, 39, 45, 51, 52, 56, 58, 59, 68) [11, 12]; HPV53 and HPV66, classified by IARC as group 2B (possibly carcinogenic), are detected by both assay panels but are not counted toward HR-HPV prevalence in this table

**Table 2:** Temporal trends in HPV prevalence across study period

Year	Region	Total	HPV_Positive	Positivity_Rate
2023	Urban	4474	546	12.20
2024	Urban	3364	589	17.51
2025	Urban	3611	645	17.86
2023	Rural	1128	135	11.97
2024	Rural	2665	305	11.44
2025	Rural	1123	169	15.05

Annual HPV prevalence and individual genotype frequencies (HPV16, HPV52, HPV58) across the 36-month study period (January 2023–December 2025), stratified by urban and rural populations. Prevalence rates with 95% Wilson CIs; temporal trends assessed using generalized estimating equations (GEE). N denotes screened women per period

**Fig. 1:**
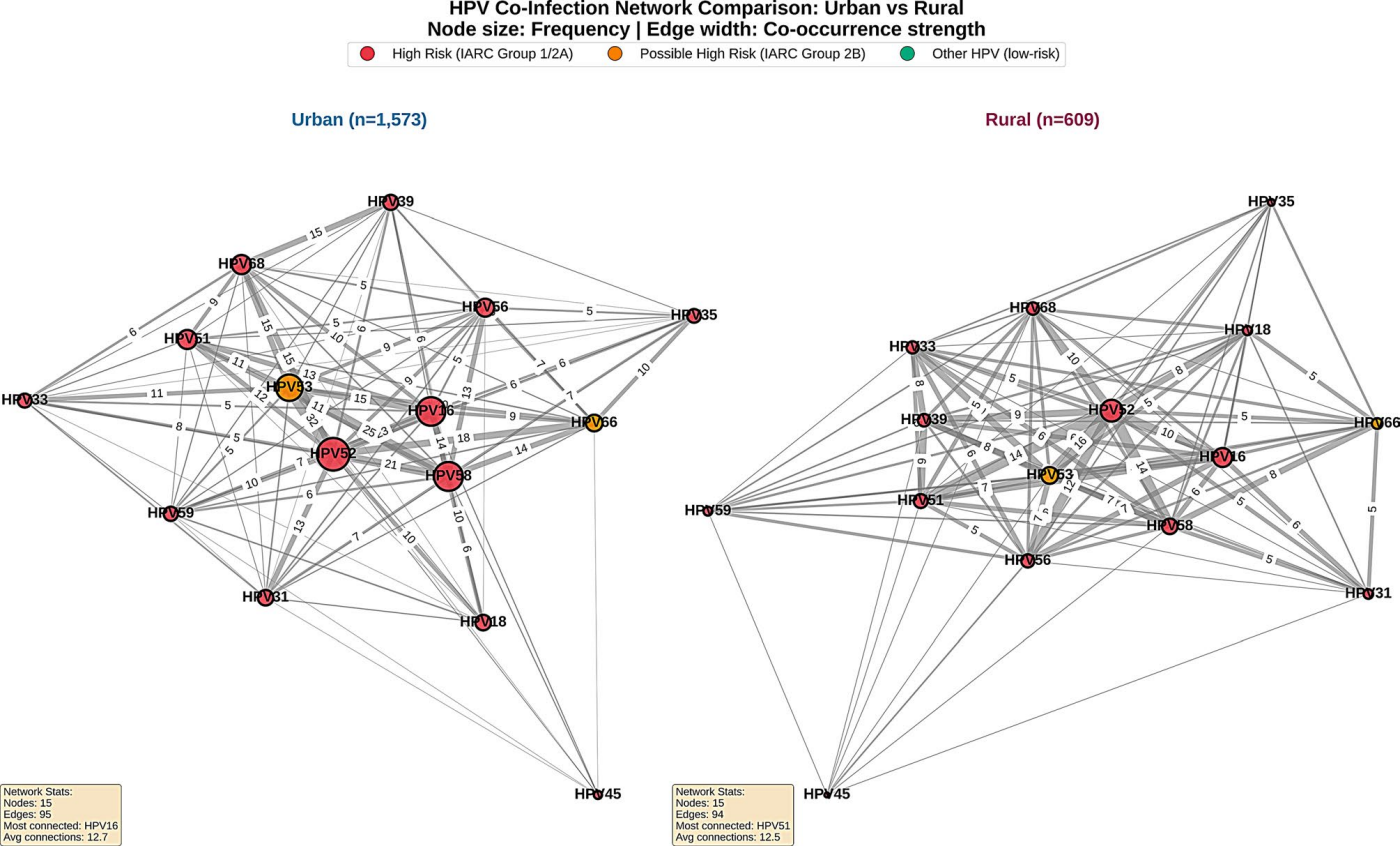
Platform-comparable HPV Co-infection network architecture: urban vs rural. HPV genotype co-infection networks restricted to the 15 high-risk genotypes detectable by both the urban and rural genotyping platforms (HPV16, 18, 31, 33, 35, 39, 45, 51, 52, 53, 56, 58, 59, 66, 68); urban *n* = 1,573 HPV-positive women, rural *n* = 609 HPV-positive women. Node size proportional to detection frequency within each cohort; node colour indicates the IARC carcinogenicity classification [11, 12]: red, IARC group 1 “carcinogenic to humans” (HPV16, 18, 31, 33, 35, 39, 45, 51, 52, 56, 58, 59) plus group 2A “probably carcinogenic” (HPV68); orange, IARC group 2B “possibly carcinogenic” (HPV53 and HPV66 within this 15-panel). Edge width proportional to co-occurrence frequency; numerical labels denote co-occurrence count for pairs with at least 5 shared cases. Mean degree (2 × edges/nodes) was 12.7 in the urban network and 12.5 in the rural network. Network statistics and top co-infection pairs are reported in Table 5; an exploratory full-panel comparison incorporating the 10 additional low-risk and probable-high-risk genotypes detectable only by the urban platform (urban: 25-type Sansure panel, 23 nodes, 199 edges, mean degree 17.3; rural unchanged) is provided in Supplementary Figure S1. Node size in both panels reflects detection frequency: HPV52, the most frequent genotype in both cohorts, has the largest node and appears in the majority of top co-infection pairs; the highest-degree node (urban: HPV16; rural: HPV51) reflects the genotype with the most distinct co-infection partners (a complementary measure of network centrality)

Temporal trends across the study period (2023–2025) were assessed using generalized estimating equations (GEE) with exchangeable correlation structure to account for within-population correlation over time, and Cochran-Armitage trend test to detect monotonic trends in proportions. For the 25 HPV genotypes analyzed, Bonferroni correction was applied to control family-wise error rate in multiple comparisons (adjusted significance level α = 0.05/25 = 0.002). Month-resolved screening volume and HPV positivity were additionally tabulated for each calendar month across the 36-month study window; monthly proportions were reported with Wilson score 95% confidence intervals (CIs), and monotonic temporal trends were tested using the non-parametric Mann–Kendall trend test on the resulting 36-point series for any-HPV and high-risk HPV (HR-HPV) positivity, separately for the urban and rural cohorts. Throughout this manuscript, HR-HPV refers to the 13 IARC Group 1/2A genotypes (HPV16, 18, 31, 33, 35, 39, 45, 51, 52, 56, 58, 59, 68) [11, 12], unless otherwise specified for the platform-comparable co-infection network analyses (Fig. 1, Supplementary Figure S1), which use the 15 platform-shared genotypes—the 13 IARC HR genotypes plus HPV53 and HPV66 (IARC Group 2B)—so that the urban and rural networks share an identical node set. Months with *N* < 30 were flagged (Low_N_Flag) and visually de-emphasised in the corresponding figure (hollow markers; marker area proportional to sqrt(N)) to prevent over-interpretation of statistically unstable monthly estimates.

Age-stratified analyses utilized five predefined age groups (<30, 30–39, 40–49, 50–59, and ≥60 years), based on established cervical cancer screening guidelines and consistent with prior epidemiological studies [24, 25].

Urban–rural differences were examined within each age stratum, and interaction effects between population type and age group were tested using logistic regression models with HPV positivity as the outcome variable. Adjusted prevalence odds ratios (aPOR) with 95% CI were calculated using logistic regression, controlling for age group (<30, 30–39, 40–49, 50–59, ≥60 years) and year of screening (2023, 2024, 2025); these covariates were selected a priori based on their known associations with HPV infection and their potential to confound urban–rural comparisons. Because outcome prevalence in this study (12.4–15.5%) exceeds 10%, aPOR estimates from logistic regression mathematically overestimate the corresponding prevalence ratio (PR). To quantify this, we performed a sensitivity analysis using modified Poisson regression with robust (HC1 sandwich) variance estimation, with the same covariate set, and report the resulting PR alongside the primary aPOR estimate.

Co-infection patterns and genotype interactions were analyzed using network analysis methods implemented in NetworkX (version 3.1). For network construction, HPV genotypes were represented as nodes with size proportional to prevalence, coloured by IARC carcinogenicity classification [11, 12]: red, IARC Group 1/2A—Group 1 “carcinogenic to humans” (HPV16, 18, 31, 33, 35, 39, 45, 51, 52, 56, 58, 59) plus Group 2A “probably carcinogenic” (HPV68); orange, IARC Group 2B “possibly carcinogenic” (HPV53 and HPV66, detectable by both panels; HPV26, 70, 73, 82 detectable only by the urban 25-type panel); and green, low-risk/non-carcinogenic genotypes (HPV6, 11, 42, 43, 81, 83). Edges connecting nodes represented co-occurrence in the same individual, with edge width proportional to co-infection frequency. The Louvain algorithm was applied to identify community structures within the networks, revealing natural groupings of frequently co-occurring genotypes.

All statistical tests were two-sided, and *p*-values < 0.05 were considered statistically significant unless otherwise specified (e.g., Bonferroni-corrected threshold for genotype comparisons). Missing data were handled using complete case analysis as the proportion of missing data was < 2%.

## Results

### Study population characteristics

**Fig. 2:**
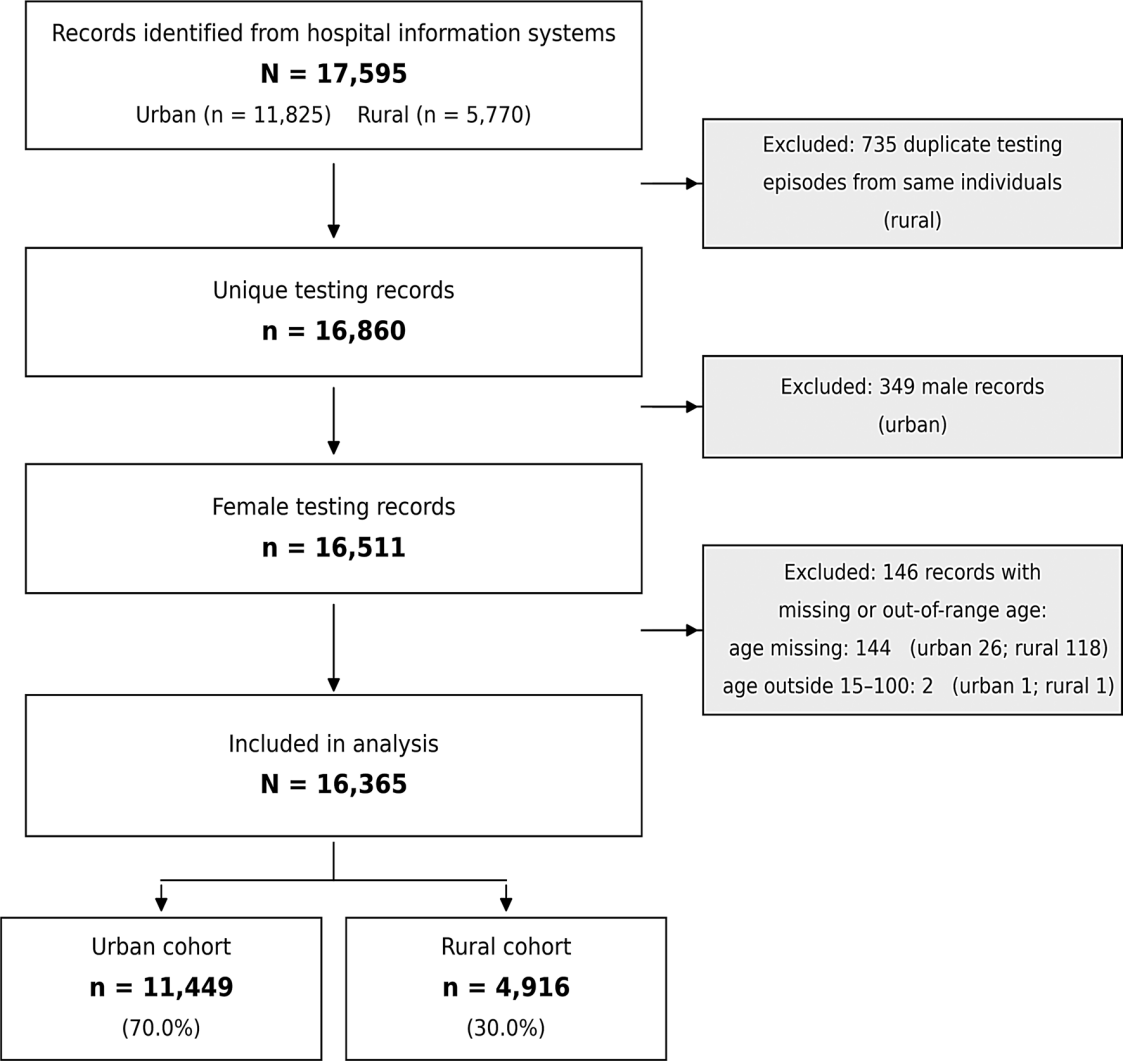
Flow diagram of study population selection. Flow of records through the study. Of 17,595 HPV testing records identified from the hospital information systems of the participating urban (*n* = 11,825) and rural (*n* = 5,770) facilities, 735 duplicate testing episodes from the same individuals were removed from the rural dataset; 349 male records (urban dataset) and 146 records were further excluded, comprising 144 records with missing age data (NaN, blank, or non-numeric values in the source datasets; urban, *n* = 26; rural, *n* = 118) and 2 records with age outside the 15–100-year eligibility window (urban, *n* = 1; rural, *n* = 1), yielding the final analytical cohort of 16,365 women (urban, *n* = 11,449; rural, *n* = 4,916). Both age-related exclusion categories were applied jointly via numeric range filtering on the age field (records with non-numeric or blank age values returned NaN under the numeric coercion step and were therefore excluded together with truly out-of-range records). HIS, hospital information system

**Fig. 3:**
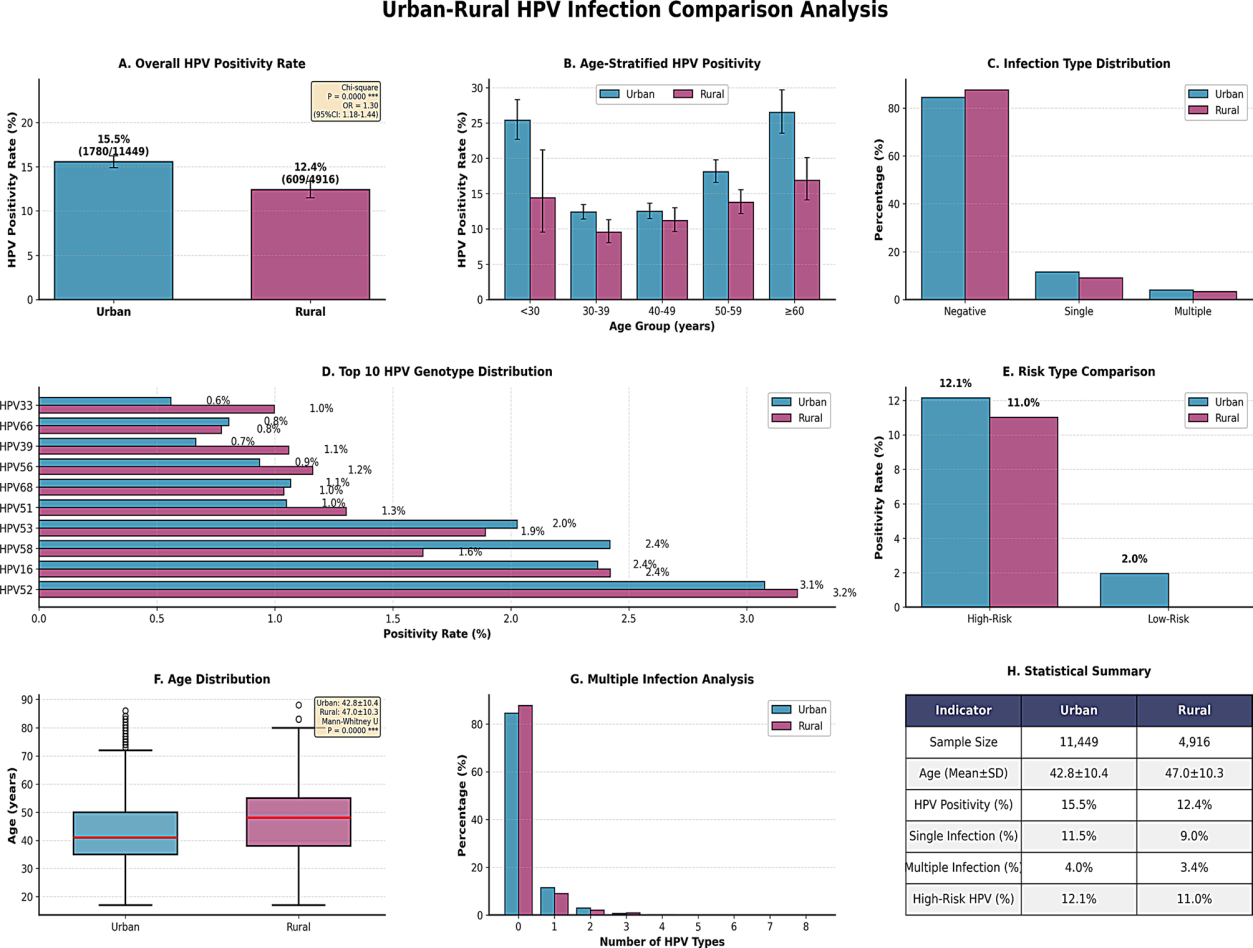
Comprehensive urban–rural HPV infection comparison dashboard. Multi-panel comparison of urban–rural HPV infection patterns (urban *n* = 11,449; rural *n* = 4,916). Panel **A**: overall HPV positivity (Chi-square test, OR with 95% CI). Panel **B**: age-stratified HPV positivity (<30, 30–39, 40–49, 50–59, ≥60 years). Panel **C**: infection type distribution (negative, single, multiple). Panel **D**: top 10 HPV genotypes by prevalence. Panel **E**: high-risk versus low-risk HPV prevalence. Panel **F**: age distribution (Mann–Whitney U test). Panel **G**: multiple infection patterns by co-infection count. Panel **H**: statistical summary table. Error bars represent 95% confidence intervals. Abbreviations: HPV, human papillomavirus; HR-HPV, high-risk HPV; OR, odds ratio; CI, confidence interval

A total of 17,595 HPV testing records were initially retrieved from the hospital information systems of the participating urban (*n* = 11,825) and rural (*n* = 5,770) facilities between January 2023 and December 2025. After removing 735 duplicate testing episodes from the same individuals in the rural dataset on the basis of personal identifiers (retaining one record per unique individual), 16,860 unique testing records remained. We further excluded 349 records corresponding to male individuals (urban dataset only) and 146 individuals with missing age data (*n* = 144; urban, *n* = 26; rural, *n* = 118) or with age outside the predefined range of 15–100 years (*n* = 2; urban, *n* = 1; rural, *n* = 1), yielding the final analytical cohort of 16,365 women, comprising 11,449 (70.0%) from urban hospitals and 4,916 (30.0%) from rural facilities in Xi’an, Shaanxi Province (Table 1). A STROBE-compliant participant flow diagram is presented in Fig. 2. The mean age was 42.8 ± 10.4 years (range: 18–85 years) in the urban cohort and 47.0 ± 10.3 years (range: 19–82 years) in the rural cohort (*p* < 0.001). Rural women were significantly older, with 45.0% aged ≥ 50 years compared with 26.4% in urban areas (Table 1, Fig. 3F). Demographic and clinical characteristics are summarised in Table 1 and Fig. 3.

### Overall HPV prevalence and urban–rural disparities

Within the platform-comparable framework (15 high-risk genotypes covered by both assays; see Methods), age-standardised HPV prevalence was 14.3% in urban (95% CI: 13.6–15.0%) versus 12.4% in rural (95% CI: 11.4–13.4%; *p* = 0.007)—a modest 1.9-percentage-point residual difference after accounting for age structure. The crude (non-age-standardised) urban positivity of 15.5% (95% CI: 14.8–16.3%) versus 12.4% rural (*p* < 0.001) is partly inflated by the 10 additional genotypes covered only by the 25-type platform (Table 3); the age-standardised, platform-comparable estimate (14.3% vs. 12.4%) is therefore taken as the primary urban–rural prevalence metric throughout the manuscript.

**Table 3:** HPV genotype distribution and urban–rural comparison

HPV Genotype	Urban n (%)	Rural n (%)	Difference (%)	p-value	OR (95% CI)
HPV6	63 (0.55%)	0 (0.00%)	−0.55%	<0.001***	54.84 (3.39–886.52)
HPV11	30 (0.26%)	0 (0.00%)	−0.26%	<0.001***	26.26 (1.61–429.59)
HPV16	271 (2.37%)	119 (2.42%)	+0.05%	0.880	0.98 (0.78–1.21)
HPV18	80 (0.70%)	28 (0.57%)	−0.13%	0.406	1.21 (0.79–1.86)
HPV26	2 (0.02%)	0 (0.00%)	−0.02%	1.000	2.15 (0.10–44.74)
HPV31	80 (0.70%)	30 (0.61%)	−0.09%	0.596	1.13 (0.75–1.72)
HPV33	64 (0.56%)	49 (1.00%)	+0.44%	0.0027**	0.56 (0.38–0.81)
HPV35	62 (0.54%)	15 (0.31%)	−0.24%	0.057	1.74 (0.99–3.03)
HPV39	76 (0.66%)	52 (1.06%)	+0.39%	0.0115*	0.62 (0.44–0.89)
HPV42	128 (1.12%)	0 (0.00%)	−1.12%	<0.001***	111.61 (6.94–1794.38)
HPV43	18 (0.16%)	0 (0.00%)	−0.16%	0.0030**	15.91 (0.96–264.12)
HPV45	22 (0.19%)	8 (0.16%)	−0.03%	0.838	1.14 (0.52–2.50)
HPV51	120 (1.05%)	64 (1.30%)	+0.25%	0.183	0.80 (0.59–1.08)
HPV52	352 (3.07%)	158 (3.21%)	+0.14%	0.673	0.95 (0.79–1.15)
HPV53	232 (2.03%)	93 (1.89%)	−0.13%	0.614	1.07 (0.84–1.36)
HPV56	107 (0.93%)	57 (1.16%)	+0.22%	0.215	0.80 (0.58–1.11)
HPV58	277 (2.42%)	80 (1.63%)	−0.79%	0.0018**	1.49 (1.16–1.92)
HPV59	67 (0.59%)	26 (0.53%)	−0.06%	0.745	1.09 (0.70–1.72)
HPV66	92 (0.80%)	38 (0.77%)	−0.03%	0.916	1.03 (0.71–1.51)
HPV68	122 (1.07%)	51 (1.04%)	−0.03%	0.938	1.02 (0.74–1.42)
HPV70	23 (0.20%)	0 (0.00%)	−0.20%	0.0004***	20.22 (1.23–333.02)
HPV73	13 (0.11%)	0 (0.00%)	−0.11%	0.0137*	11.61 (0.69–195.30)
HPV81	94 (0.82%)	0 (0.00%)	−0.82%	<0.001***	81.83 (5.08–1318.20)
HPV82	24 (0.21%)	0 (0.00%)	−0.21%	0.0004***	21.09 (1.28–346.81)
HPV83	4 (0.03%)	0 (0.00%)	−0.03%	0.324	3.87 (0.21–71.82)

Prevalence of 25 HPV genotypes (15 high-risk, 3 probable high-risk, 7 low-risk per established Chinese clinical convention; IARC carcinogenicity grouping detailed in introduction) by setting. Urban–rural comparisons by chi-square or Fisher’s exact test (when expected cell counts < 5); Bonferroni-adjusted significance threshold α = 0.05/25 = 0.002. Rural counts of 0 (0.00%) for HPV6, HPV11, HPV26, HPV42, HPV43, HPV70, HPV73, HPV81, HPV82, and HPV83 reflect non-coverage by the 15-type rural assay (see Methods); *p*-values and odds ratios for these 10 genotypes are reported for descriptive completeness only and should not be interpreted as evidence of true urban–rural prevalence differences

The crude HPV positivity rate was 15.5% (95% CI: 14.8–16.3%, 1,780/11,449) in urban populations and 12.4% (95% CI: 11.4–13.4%, 609/4,916) in rural populations (χ^2^ = 21.45, *p* < 0.001) (Fig. 3A, Table 1), consistent with the age-standardised comparison (14.3% vs. 12.4%; *p* = 0.007) and confirming significantly higher HPV burden in urban settings after controlling for age distribution. After adjustment for age group and year of screening using logistic regression, the overall adjusted prevalence odds ratio (aPOR) for urban versus rural was 1.42 (95% CI: 1.28–1.57; *p* < 0.001); a pre-specified sensitivity analysis using modified Poisson regression with robust (HC1) variance estimation yielded a prevalence ratio (PR) of 1.34 (95% CI: 1.23–1.47; *p* < 0.001), with the modest difference between aPOR and PR reflecting the expected mathematical overestimation when outcome prevalence exceeds 10%; the direction, statistical significance, and substantive interpretation of the urban–rural disparity were therefore robust to choice of estimator. When stratified by IARC carcinogenicity classification (HR-HPV defined as the 13 IARC Group 1/2A genotypes: HPV16, 18, 31, 33, 35, 39, 45, 51, 52, 56, 58, 59, 68 [11, 12]), high-risk HPV prevalence showed similar urban–rural differences (urban: 12.1%, 95% CI: 11.5–12.8%, 1,391/11,449; rural: 11.0%, 95% CI: 10.1–12.0%, 542/4,916; *p* = 0.044) (Fig. 3E, Table 1).

The distribution of infection types differed between populations (Fig. 3C, Table 1). Single HPV infections were more common in urban areas (11.5%) compared to rural areas (9.0%, *p* < 0.001), while multiple infections (simultaneous detection of ≥ 2 genotypes) occurred in 4.0% of urban women versus 3.4% of rural women (*p* = 0.087).

### Temporal trends in HPV prevalence

**Fig. 4:**
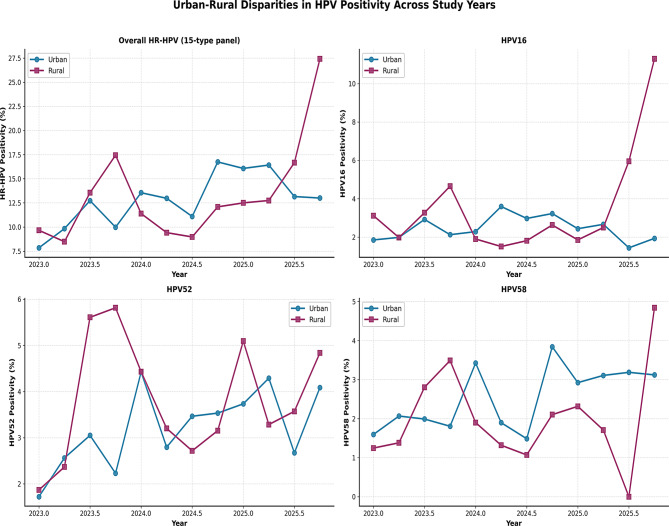
Temporal trends in HPV prevalence by geographic setting. Temporal trends in HPV prevalence across the 36-month study period (January 2023–December 2025), stratified by urban (*n* = 11,449) and rural (*n* = 4,916) populations. The top-left panel shows overall high-risk HPV (HR-HPV) prevalence, defined as detection of any of the 13 IARC group 1/2A high-risk genotypes [11, 12] (HPV16, 18, 31, 33, 35, 39, 45, 51, 52, 56, 58, 59, 68); remaining panels show HPV16, HPV52, and HPV58 trends. Numerical data and generalized estimating equation (GEE) regression statistics are reported in Table 2. Abbreviations: HPV, human papillomavirus; HR-HPV, high-risk HPV; IARC, International Agency for Research on Cancer; GEE, generalized estimating equation

**Fig. 5:**
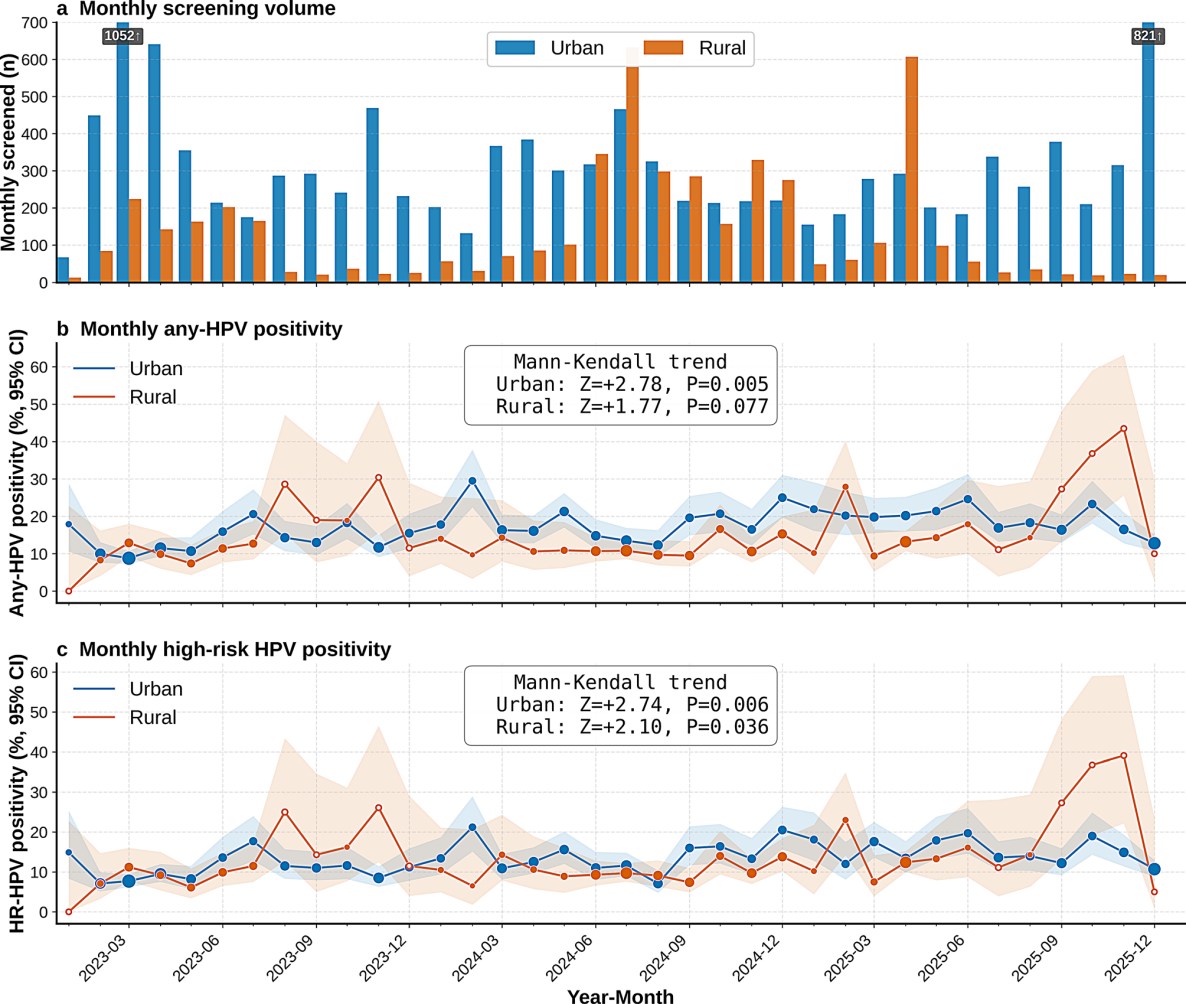
Monthly screening volume and HPV positivity, urban–rural (2023–2025). Tri-panel month-resolved view of the cervical screening cohort across the 36-month study window. (**a**) Monthly count of women screened, urban (blue) versus rural (vermilion); for typographic clarity the y-axis is capped at *n* = 700 with the two outlying months annotated above the cap with the actual count followed by an upward arrow (March 2023 urban: 1,052↑; December 2025 urban: 821↑). (**b**) Monthly any-HPV positivity (%) with Wilson 95% confidence interval ribbons. (**c**) Monthly high-risk HPV (HR-HPV) positivity (%) with Wilson 95% CI. In panels (**b**) and (**c**), filled circles denote months with *N* ≥ 30 (statistically stable), hollow circles denote months with *N* < 30 (unstable estimates, not for single-point interpretation), and marker area is proportional to sqrt(N). Mann–Kendall tests for monotonic temporal trend are reported in each rate panel as Z statistic with two-sided *p* value. Both urban and rural cohorts show a statistically significant upward trend in HR-HPV positivity (urban Z = +2.74, *p* = 0.006; rural Z = +2.10, *p* = 0.036). Numerical data and Low_N flags are provided in Supplementary table S4. Abbreviations: HPV, human papillomavirus; HR-HPV, high-risk HPV; CI, confidence interval; N, monthly sample size

Temporal trend analysis across the 36-month study period (January 2023 to December 2025) revealed dynamic patterns in HPV prevalence (Fig. 4, Table 2). Overall high-risk HPV prevalence in urban areas ranged from 7.9% to 16.7% across six-month intervals, with notable fluctuations but no significant linear trend (GEE β = 0.0012, *p* = 0.342). Rural high-risk HPV prevalence ranged from 9.5% to 27.1%, showing greater temporal variability but also no significant trend (GEE β = 0.0023, *p* = 0.428). Month-resolved screening volumes and any-HPV/HR-HPV positivity, with Wilson 95% confidence intervals for each monthly proportion and Mann–Kendall non-parametric tests for monotonic temporal trend, are presented in Fig. 5 and Supplementary Table S4; Mann–Kendall testing identified a statistically significant monotonic increase in HR-HPV positivity across the 36-month observation window in both populations (urban Z = +2.74, *p* = 0.006; rural Z = +2.10, *p* = 0.036), and a parallel significant increase in any-HPV positivity in the urban population (Z = +2.78, *p* = 0.005), with the rural any-HPV trend approaching but not reaching significance (Z = +1.77, *p* = 0.077). The catch-up screening interpretation of these temporal patterns is developed in the Discussion (Temporal Trends and COVID-19 Impact).

Examination of individual high-risk genotypes revealed distinct temporal patterns (Fig. 4). HPV16 prevalence showed remarkable stability in urban areas (range: 1.5–3.5%) but exhibited substantial fluctuation in rural areas (range: 1.5–10.9%), with a notable spike in the most recent period (July-December 2025). HPV52, the most prevalent genotype, demonstrated relative stability in urban settings (range: 1.8–4.4%) while showing periodic peaks in rural areas. HPV58 prevalence fluctuated between 1.6–3.9% in urban areas and 0–4.7% in rural areas, with a pronounced rural peak in the latest measurement period.

### High-risk HPV genotype distribution

**Fig. 6:**
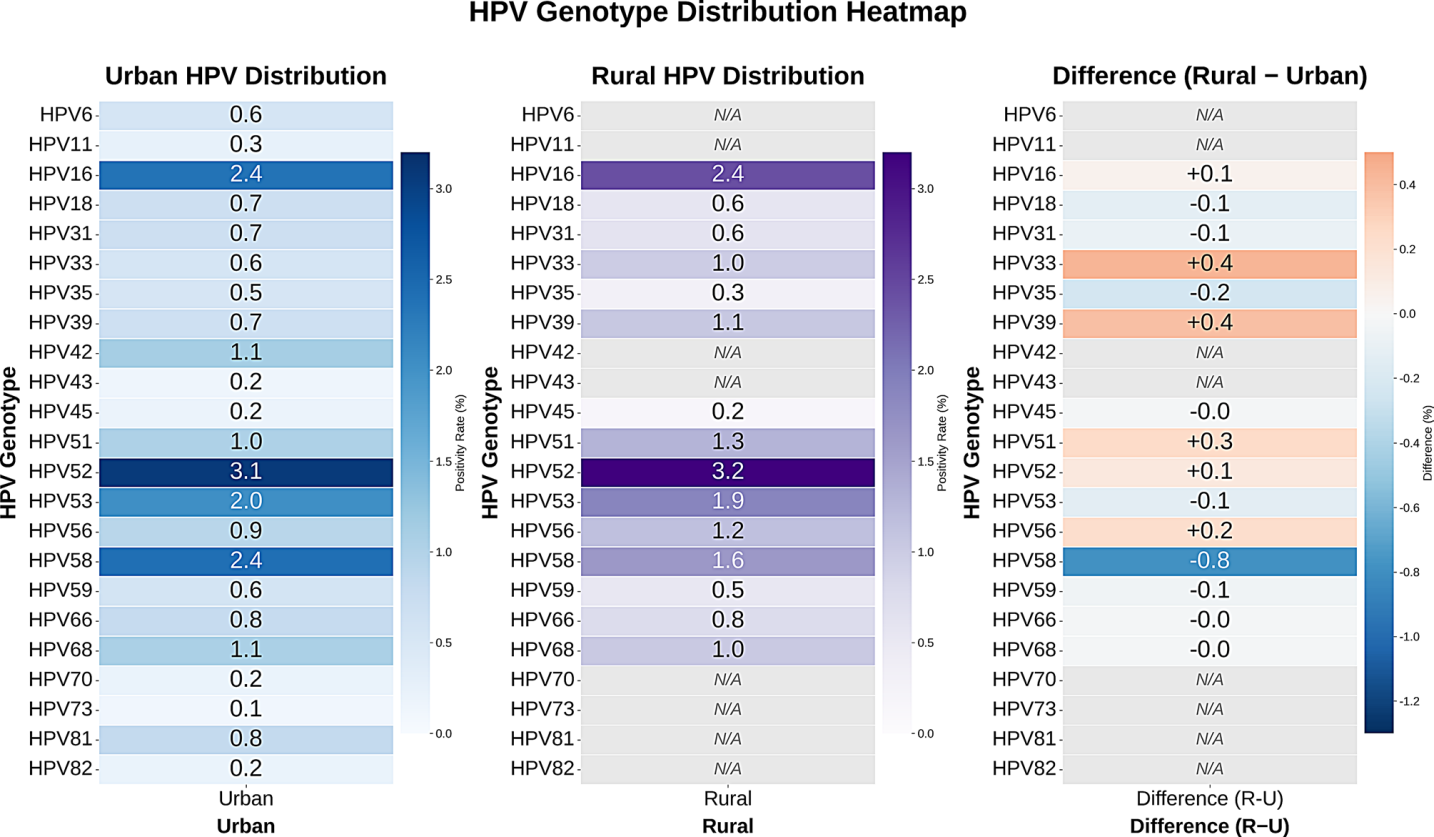
Urban–rural HPV genotype distribution heatmap. Three-panel heatmap of HPV genotype prevalence (urban *n* = 11,449; rural *n* = 4,916): urban distribution (left), rural distribution (middle), and rural − urban difference (right). Color intensity proportional to prevalence (darker indicates higher). Genotypes detectable only by the urban 25-type platform (HPV6, 11, 26, 42, 43, 70, 73, 81, 82, 83) are shown as N/A in the rural panel. Statistical comparisons (chi-square or Fisher’s exact, with Bonferroni adjustment for multiple genotypes) and adjusted *p*-values are reported in Table 3. Abbreviations: HPV, human papillomavirus; N/A, not assessed (genotype outside the rural detection panel)

Among the 15 high-risk HPV genotypes examined, HPV52 was the most prevalent type in both urban (3.1%, 95% CI: 2.8–3.5%) and rural (3.2%, 95% CI: 2.6–3.8%) populations, followed by HPV16 (urban: 2.4%, 95% CI: 2.1–2.8%; rural: 2.4%, 95% CI: 1.9–3.0%) and HPV58 (urban: 2.4%, 95% CI: 2.1–2.8%; rural: 1.6%, 95% CI: 1.2–2.1%) (Figs. 3D, 6, Table 3). The top 10 most prevalent HPV genotypes in descending order were: HPV52, HPV16, HPV58, HPV53, HPV51, HPV39, HPV68, HPV66, HPV33, and HPV6.

Significant urban–rural differences in genotype-specific prevalence were observed for several types (Fig. 6, Table 3). Urban populations demonstrated significantly higher prevalence of HPV58 (2.4% vs. 1.6%, *p* = 0.002), HPV42 (1.1% vs. 0%, *p* < 0.001), and HPV81 (0.8% vs. 0%, *p* < 0.001). In contrast, rural populations showed higher prevalence of HPV33 (1.0% vs. 0.6%, *p* = 0.003) and HPV39 (1.1% vs. 0.7%, *p* = 0.012). For the most prevalent genotype HPV52, no significant difference was observed between urban and rural settings (3.1% vs. 3.2%, *p* = 0.673). These patterns remained significant after Bonferroni correction for multiple comparisons (adjusted α = 0.002).

The five most common high-risk genotypes (HPV52, HPV16, HPV58, HPV53, HPV51) collectively accounted for 58.1% of all HPV infections in urban areas and 62.7% in rural areas.

### Age-specific HPV prevalence patterns

**Fig. 7:**
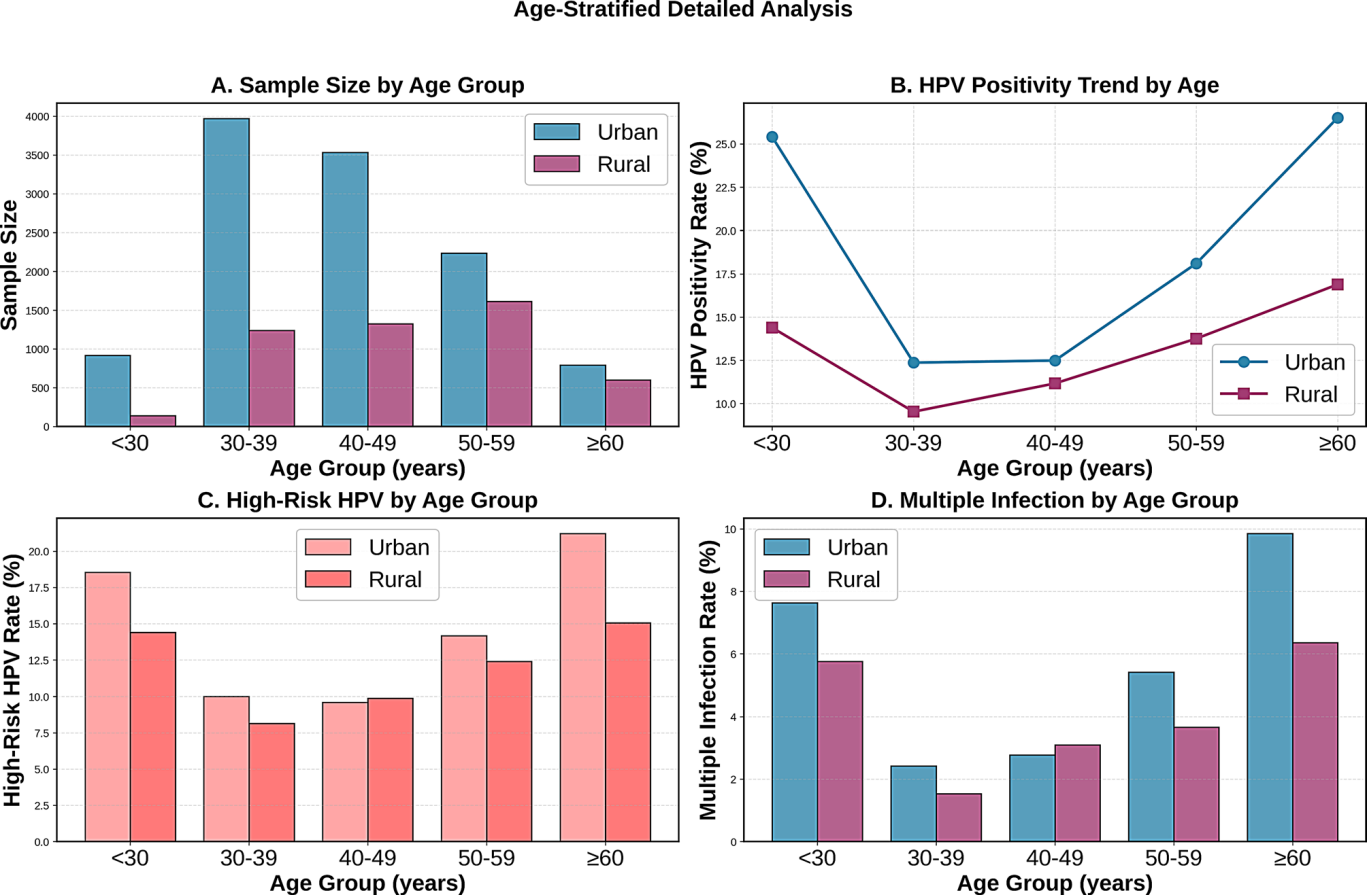
Age-stratified HPV prevalence and infection patterns. Four-panel age-stratified analysis of HPV infection (urban *n* = 11,449; rural *n* = 4,916; five age groups: <30, 30–39, 40–49, 50–59, ≥60 years). Panel **A**: sample size distribution by age group. Panel **B**: age-specific HPV positivity. Panel **C**: high-risk HPV (HR-HPV) prevalence by age group. Panel **D**: multiple infection rates by age group. Statistical comparisons and adjusted prevalence odds ratios are reported in Table 4. Abbreviations: HPV, human papillomavirus; HR-HPV, high-risk HPV

Age-specific analysis revealed distinct distribution patterns between urban and rural populations (Figs. 3B, 7, Table 4). Urban women demonstrated a characteristic U-shaped age distribution with two distinct peaks: the highest prevalence occurred in the <30 years age group (25.4%, 95% CI: 22.7–28.3%), followed by a gradual decline through middle age (30–39 years: 12.4%, 95% CI: 11.4–13.5%; 40–49 years: 12.5%, 95% CI: 11.5–13.6%), and a secondary peak in the ≥60 years age group (26.5%, 95% CI: 23.5–29.7%) (Fig. 7B).

**Table 4:** Age-stratified HPV prevalence and infection patterns

Age Group	Urban n	Rural n	Urban HPV+(%)	Rural HPV+(%)	Urban HR-HPV(%)	Rural HR-HPV(%)	Urban Multi(%)	Rural Multi(%)
<30	917	139	25.4%	14.4%	18.5%	14.4%	7.6%	5.8%
30–39	3,971	1,239	12.4%	9.5%	10.0%	8.2%	2.4%	1.5%
40–49	3,532	1,326	12.5%	11.2%	9.6%	9.9%	2.8%	3.1%
50–59	2,237	1,614	18.1%	13.8%	14.2%	12.4%	5.4%	3.7%
≥60	792	598	26.5%	16.9%	21.2%	15.1%	9.8%	6.4%

HPV infection metrics by age group (<30, 30–39, 40–49, 50–59, ≥60 years) and setting. Urban–rural differences within each stratum tested by chi-square; age-by-setting interaction tested by logistic regression with HPV positivity as outcome, adjusted for year of screening; aPOR and modified-Poisson PR sensitivity estimates reported in results

**Fig. 8:**
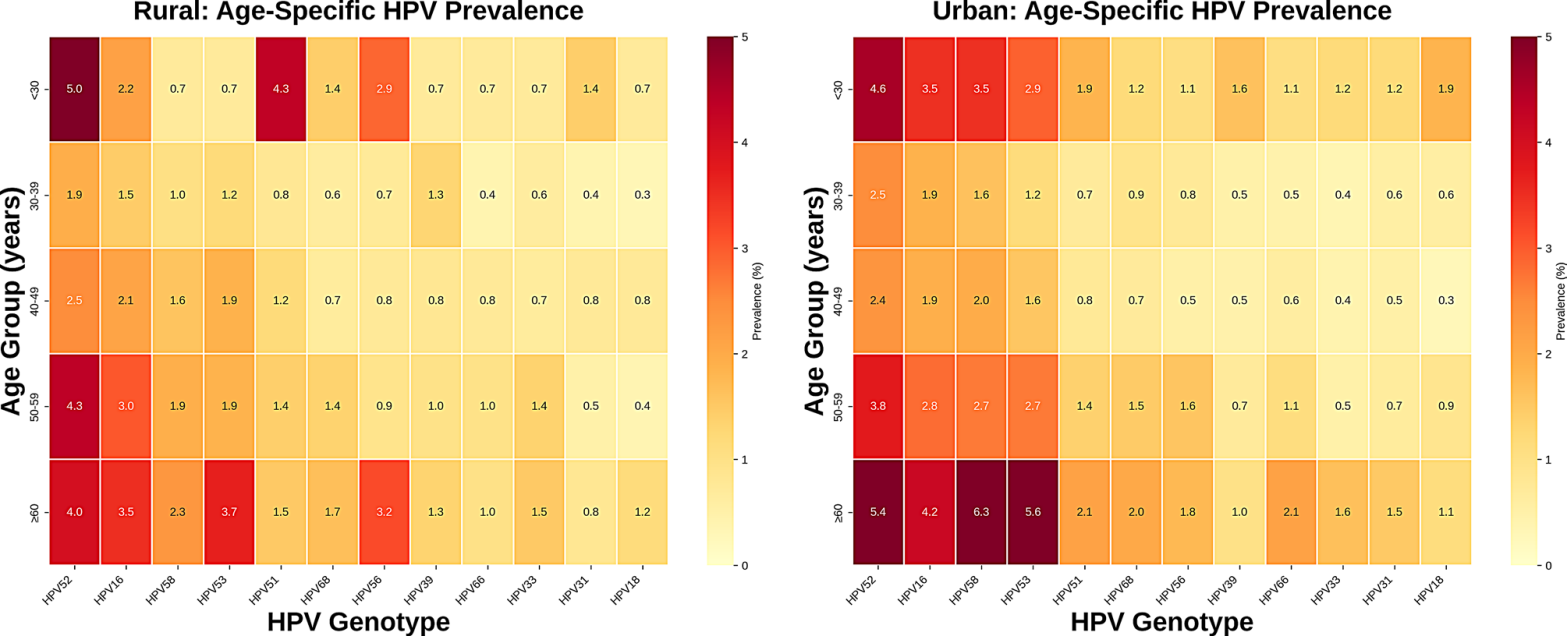
Age-specific HPV genotype prevalence heatmaps. Dual heatmap of age-stratified prevalence of the 12 HPV genotypes detected by both urban and rural genotyping platforms with overall prevalence ≥ 0.3% (HPV52, 16, 58, 53, 51, 68, 56, 39, 66, 33, 31, 18), shown for rural (*n* = 4,916; left) and urban (*n* = 11,449; right) populations across five age groups (<30, 30–39, 40–49, 50–59, ≥60 years). Darker color indicates higher prevalence; numerical values annotated within each cell. Complete numerical data including 95% confidence intervals are in table 6. Abbreviations: HPV, human papillomavirus

In contrast, rural women showed a different pattern with HPV prevalence gradually increasing with age, from 14.4% in the <30 years group to 16.9% in the ≥60 years age group (Fig. 7B, Table 4). The age-by-setting interaction was statistically significant (P for interaction < 0.001), indicating fundamentally different age-related patterns between urban and rural populations. The age-stratified prevalence of the 12 most common HPV genotypes detected by both urban and rural genotyping platforms is presented in Table 6 and visualised as dual-population heatmaps in Fig. 8.

### Multiple HPV infections and co-infection patterns

Multiple HPV infections (defined as simultaneous detection of two or more HPV genotypes) were detected in 628 women, representing 26.3% of all HPV-positive cases (Fig. 3G). Urban women had higher multiple infection rates (4.0%, 95% CI: 3.6–4.5%, 463/11,449) compared to rural women (3.4%, 95% CI: 2.8–4.0%, 165/4,916; χ^2^ = 2.93, *p* = 0.087). Among HPV-positive individuals, 26.0% of urban cases and 27.1% of rural cases involved multiple genotypes.

The distribution of co-infection burden showed that most multiple infections involved two genotypes (urban: 86.8%, rural: 87.3%), followed by three genotypes (urban: 10.4%, rural: 9.7%), and ≥4 genotypes (urban: 2.8%, rural: 3.0%) (Fig. 3G). The maximum number of simultaneous genotypes detected was 8 in one urban case and 7 in one rural case.

Network analysis of co-infection patterns was performed within the platform-comparable framework, restricting both networks to the 15 high-risk genotypes detectable by both detection panels (HPV16, 18, 31, 33, 35, 39, 45, 51, 52, 53, 56, 58, 59, 66, 68; see Methods) so that urban and rural networks share an identical node set and are directly density-comparable (Fig. 1, Table 5). Within this framework, the urban network (*n* = 1,573 HPV-positive women restricted to detection of these 15 genotypes) comprised 15 nodes with 95 edges, while the rural network (*n* = 609 HPV-positive women) comprised 15 nodes with 94 edges. Mean degree (2E/N) was nearly identical between settings—12.7 (urban; 2 × 95 / 15) versus 12.5 (rural; 2 × 94 / 15; ratio 1.02)—indicating that, when restricted to the platform-shared 15 high-risk genotypes, urban and rural co-infection networks exhibit comparable overall density.

**Table 5:** Platform-comparable HPV Co-infection network statistics: urban vs rural

A. HPV Genotype Frequency Comparison				
HPV Type	Urban (n)	Urban (%)	Rural (n)	Rural (%)
HPV52	352	22.4	158	25.9
HPV58	277	17.6	80	13.1
HPV16	271	17.2	119	19.5
HPV53	232	14.7	93	15.3
HPV68	122	7.8	51	8.4
HPV51	120	7.6	64	10.5
HPV56	107	6.8	57	9.4
HPV66	92	5.8	38	6.2
HPV18	80	5.1	28	4.6
HPV31	80	5.1	30	4.9
HPV39	76	4.8	52	8.5
HPV59	67	4.3	26	4.3
HPV33	64	4.1	49	8.0
HPV35	62	3.9	15	2.5
HPV45	22	1.4	8	1.3
**B. Network Statistics Comparison**				
**Metric**	**Urban**	**—**	**Rural**	**—**
Total Positive Cases	1,573	—	609	—
Network Nodes	15	—	15	—
Network Edges	95	—	94	—
**C. Top Co-Infection Pairs**				
**Rank**	**Urban Pair**	**Count**	**Rural Pair**	**Count**
1	HPV52+HPV53	32	HPV52+HPV53	16
2	HPV58+HPV53	25	HPV51+HPV52	14
3	HPV16+HPV52	23	HPV52+HPV58	14
4	HPV52+HPV58	21	HPV52+HPV56	12
5	HPV52+HPV66	18	HPV16+HPV52	10
6	HPV16+HPV53	15	HPV16+HPV53	10
7	HPV39+HPV68	15	HPV52+HPV68	10
8	HPV52+HPV68	15	HPV33+HPV53	9
9	HPV68+HPV53	15	HPV39+HPV51	9
10	HPV16+HPV58	14	HPV39+HPV52	9

Platform-comparable HPV co-infection network statistics. Network analysis restricted to the 15 high-risk genotypes detectable by both detection panels (HPV16, 18, 31, 33, 35, 39, 45, 51, 52, 53, 56, 58, 59, 66, 68); urban *n* = 1,573 HPV-positive women, rural *n* = 609 HPV-positive women. Section a reports HPV genotype frequency among HPV-positive cases within each cohort; Section B reports network-level summary statistics (total HPV-positive cases, network nodes, network edges); Section C lists the top 10 co-infection pairs by co-occurrence count. Mean degree (2 × edges/nodes) was 12.7 in the urban network and 12.5 in the rural network. An exploratory full-panel comparison (urban: 25-type Sansure panel; rural: 15-type panel) is provided in Supplementary Figure S1 with companion data in Supplementary table S3

Across both networks, HPV52+HPV53 was the most frequent co-infection pair (urban: 32 cases; rural: 16); the remaining top-five urban pairs were HPV58+HPV53 (25), HPV16+HPV52 (23), HPV52+HPV58 (21), and HPV52+HPV66 (18), while the remaining top-five rural pairs were HPV51+HPV52 (14), HPV52+HPV58 (14), HPV52+HPV56 (12), and HPV16+HPV52 (10) (Table 5C, Fig. 1). HPV52 appeared in four of the top-five urban pairs and in all five top-five rural pairs, identifying it as the central network hub in both settings. The near-identical platform-comparable mean degree (12.7 vs 12.5) argues against a substantive urban–rural difference in HR-HPV co-infection ecology once detection-panel asymmetry is removed. To complement this primary platform-comparable analysis, an exploratory full-panel comparison (urban: 25-type Sansure panel, *n* = 1,780 HPV-positive, 23 nodes, 199 edges, mean degree 17.3; rural: 15-type panel as above, mean degree 12.5; ratio 1.38) is provided in Supplementary Figure S1 (with companion data in Supplementary Table S3). The apparent higher urban density observed in the full-panel view is largely attributable to the 10 additional low-risk and probable-high-risk genotypes detectable only by the urban platform and is therefore not directly density-comparable; clinically, a higher mean degree, where it occurs, implies more frequent multi-genotype co-infections that may complicate triage and risk stratification based on any single genotype.

## Summary of statistical comparisons

A comprehensive statistical comparison of key indicators between urban and rural populations is presented in Fig. 3H. Urban populations demonstrated significantly higher overall HPV positivity (age-standardised: 14.3% vs. 12.4%, *p* = 0.007; crude: 15.5% vs. 12.4%, *p* < 0.001), higher single infection rates (11.5% vs. 9.0%, *p* < 0.001), and higher high-risk HPV prevalence (12.1% vs. 11.0%, *p* = 0.044). Multiple infection rates showed a trend toward higher urban prevalence (4.0% vs. 3.4%, *p* = 0.087).

**Table 6:** Age-Specific HPV Genotype Prevalence by Population. Age-stratified prevalence (%) of the 12 most common HPV genotypes for rural (Part 1) and urban (Part 2) populations across five age groups (<30, 30–39, 40–49, 50–59, ≥60 years), ordered by overall prevalence within each population

Part 1 — Rural population												
**Age Group**	**HPV52**	**HPV16**	**HPV58**	**HPV53**	**HPV51**	**HPV68**	**HPV56**	**HPV39**	**HPV66**	**HPV33**	**HPV31**	**HPV18**
<30	5.04%	2.16%	0.72%	0.72%	4.32%	1.44%	2.88%	0.72%	0.72%	0.72%	1.44%	0.72%
30–39	1.94%	1.45%	1.05%	1.21%	0.81%	0.65%	0.73%	1.29%	0.40%	0.65%	0.40%	0.32%
40–49	2.49%	2.11%	1.58%	1.89%	1.21%	0.68%	0.75%	0.83%	0.75%	0.68%	0.75%	0.75%
50–59	4.34%	3.04%	1.92%	1.86%	1.43%	1.36%	0.93%	0.99%	0.99%	1.36%	0.50%	0.37%
≥60	4.01%	3.51%	2.34%	3.68%	1.51%	1.67%	3.18%	1.34%	1.00%	1.51%	0.84%	1.17%
**Part 2 — Urban population**												
<30	4.58%	3.49%	3.49%	2.94%	1.85%	1.20%	1.09%	1.64%	1.09%	1.20%	1.20%	1.85%
30–39	2.49%	1.89%	1.64%	1.16%	0.71%	0.93%	0.76%	0.50%	0.45%	0.35%	0.58%	0.60%
40–49	2.38%	1.93%	1.98%	1.56%	0.76%	0.71%	0.51%	0.48%	0.62%	0.42%	0.54%	0.28%
50–59	3.76%	2.82%	2.68%	2.68%	1.39%	1.48%	1.56%	0.72%	1.12%	0.49%	0.67%	0.89%
≥60	5.43%	4.17%	6.31%	5.56%	2.15%	2.02%	1.77%	1.01%	2.15%	1.64%	1.52%	1.14%

## Discussion

### Summary of Principal findings

This large-scale population-based survey documents urban–rural differences in HPV infection patterns in Northwest China across overall prevalence, genotype composition, infection complexity, and age-specific distribution. Within the platform-comparable, age-standardised framework, the residual urban–rural gap is modest (1.9 percentage points; 14.3% vs. 12.4%, *p* = 0.007), and detection-platform heterogeneity should be considered when interpreting crude differences. Three overarching patterns emerge. First, the urban excess in HPV positivity paradoxically reverses the direction expected from global surveillance data, implicating healthcare-seeking behaviour and screening programme design as mediating factors distinct from biological exposure risk. Second, HPV52 and HPV58 collectively dominate the infection landscape across both settings—a genotype profile that diverges substantially from the HPV16/18-centric paradigm underpinning current vaccine formulations, with direct consequences for population-level vaccine effectiveness in this region.

Third, within the rural sub-cohort, the histopathological follow-up rate (11.5%; *n* = 70/609 HPV-positive rural women) falls far below the WHO-recommended minimum of 70%, exposing a critical gap between screening access and care completion that may attenuate the public health benefit of organised screening programmes; matched urban follow-up cascade data were not available for the present analysis, which precludes formal urban–rural cascade comparison and is identified as a priority for subsequent matched-cohort studies. Taken together, these findings challenge uniform national screening policy frameworks and point to the need for setting-specific intervention strategies that address not only detection but also cascade-of-care continuity, particularly in rural settings where the documented gap is most pronounced.

### Comparison, contrast, and contextualisation with existing literature

Our findings align with recent Chinese nationwide surveillance data, but reveal important geographic variations. The higher urban prevalence contrasts with global patterns where rural areas in developing countries typically show higher infection rates [49]. This reversal likely reflects China’s unique urbanization trajectory and healthcare accessibility differences rather than intrinsic epidemiological differences.

The HPV52 predominance—23.4% of HPV-positive cases under the platform-comparable framework (510/2,182) and 21.3% in the full-panel crude cohort (510/2,389)—is consistent with East Asian epidemiological patterns, differing markedly from Western populations where HPV16 accounts for 30–40% of infections [50]. These genotype distribution differences have profound implications for HPV vaccination strategies, as current bivalent or quadrivalent vaccines targeting HPV16/18 would theoretically prevent a substantially smaller fraction of infections in our population than the approximately 30–40% prevention coverage estimated in Western populations, where HPV16 alone accounts for the majority of HPV-positive cases [50]. Population-level impact studies have demonstrated that nine-valent vaccination including HPV52 could substantially improve cervical cancer prevention in Asian populations [23, 51].

The age-specific patterns observed in our urban population were broadly similar to bimodal distributions reported in some high-income settings, in which younger-age peaks have been associated with the period around sexual debut and older-age peaks have been variously linked to immune senescence, latent infection reactivation, or birth-cohort effects. Conversely, the monotonic age increase observed in our rural population is consistent with patterns reported in settings with less organised screening, in which cumulative exposure may accumulate throughout the lifespan without interruption by screening-driven detection [25, 50]. A critical methodological observation is warranted when comparing our findings with published Chinese and international HPV surveillance studies. Most prior studies relied on convenience samples from gynaecological outpatient clinics rather than population-based screening programmes, introducing selection bias towards symptomatic or higher-risk women that likely inflates reported prevalence estimates relative to our study population [18, 49]. Furthermore, geographic heterogeneity within China—particularly differences in ethnic composition, climate, and urbanisation trajectory between Northwest, Southeast, and Central provinces—limits direct numerical comparability across studies [5, 18]. Our findings should therefore be interpreted as regionally representative of Northwest China rather than nationally generalisable.Connecting these findings to China’s national cervical cancer elimination agenda, our data document substantial HPV burden in both urban (younger-age peak) and rural (cumulative-age increase) populations alongside a critical rural cascade-of-care gap (11.5% pathological follow-up); matched urban cascade-of-care data were not available for the present analysis, so the relative magnitude of the urban cascade gap (if any) cannot be directly compared. The WHO global strategy [24] targets of 70% screening coverage and 90% treatment coverage of women with positive screening cannot be assumed to be achievable through uniform programme expansion alone; rural access and cascade-of-care barriers documented here, together with prospective matched-cohort evaluation of urban–rural cascade indicators identified as a research priority, will both be required to achieve the 90–70-90 targets within the projected timeframe [23, 24].

## Mechanistic interpretation

### Detection bias and healthcare access

In the absence of individual-level data on sexual behaviour or other established HPV risk factors in this dataset, the higher urban HPV positivity is most plausibly attributable to a combination of detection-platform asymmetry, age-structure differences, and differential healthcare access between the two settings, rather than to a true difference in underlying HPV exposure. Urban facilities in Xi’an provided either the comprehensive 25-type assay or the 15-type high-risk-only assay, while rural facilities used the 15-type assay exclusively, contributing to the apparent urban excess in crude positivity that attenuated to a 1.9-percentage-point residual difference within the platform-comparable, age-standardised framework (14.3% vs. 12.4%; *p* = 0.007).

Supporting this interpretation, urban specimens in our study were tested with either the comprehensive 25-type HPV genotyping platform or the 15-type high-risk-only assay, while rural specimens were uniformly tested with the 15-type panel. Detection-platform heterogeneity therefore primarily affects urban detection of the 10 genotypes covered only by the 25-type panel—including detection events in women whose only HPV infection is one of these non-high-risk genotypes—and contributes to the apparent urban excess in crude HPV positivity (15.5% vs. 12.4%). After restricting comparisons to the 15 high-risk genotypes covered by both platforms and applying age standardisation, a modest 1.9-percentage-point residual urban–rural difference remained (14.3% vs. 12.4%, *p* = 0.007), indicating that platform heterogeneity contributes to but does not fully account for the apparent urban–rural difference in HR-HPV burden.

### Age-specific immune dynamics

The U-shaped urban age distribution reflects complex interactions between behavioral factors, immune surveillance, and cohort effects. The initial peak in women < 25 years corresponds to recent sexual debut and higher partner turnover rates. The subsequent decline in 25–34 year-olds likely represents effective immune clearance, consistent with cohort evidence that the majority of incident HPV infections in young immunocompetent women regress within two years through cell-mediated immune control [10].

The secondary peak in women ≥ 55 years may involve multiple mechanisms: immune senescence reducing HPV clearance efficiency, reactivation of latent infections under reduced immune surveillance, and cohort effects as these women reached sexual maturity before widespread awareness of cervical cancer prevention. The monotonic rural age increase suggests that cumulative exposure risk predominates, with limited effect of immune clearance, potentially reflecting lower baseline immune function and limited access to regular screening.

### HPV52 predominance and viral fitness

The HPV52 predominance in East Asian populations may reflect viral genetic factors conferring competitive advantages in specific host genetic backgrounds. Recent lineage and variant analyses of HPV52 and HPV58 among Chinese women have characterised distinct sublineages circulating in East Asia, with sequence variants in the E6/E7 region that may modulate oncogene activity relative to lineages predominating in Europe and the Americas [19]. Together with HLA-typing evidence that common East Asian HLA haplotypes generate weaker HPV52-specific T-cell responses than HPV16-specific responses, these viral and host factors plausibly co-determine the selective geographic predominance of HPV52 observed in our cohort.

### Multiple infection dynamics

The slightly higher urban multiple infection rate (4.0% vs. 3.4%, *p* = 0.087, Table 1) did not reach statistical significance, but the directional difference may reflect greater exposure diversity in urban settings rather than immune dysfunction. The notably younger mean age of urban women (42.8 ± 10.4 years vs. 47.0 ± 10.3 years, *p* < 0.001) may partially account for the urban excess in both overall HPV positivity and multiple infection rates, given the well-established association between younger age and higher HPV acquisition risk; this demographic difference, alongside lifestyle and healthcare-access factors, should be considered when interpreting urban–rural disparities in this study. Within the platform-comparable framework (15 platform-shared high-risk genotypes; Fig. 1), urban and rural co-infection network densities were nearly identical (mean degree 12.7 vs 12.5; ratio 1.02), with HPV52 emerging as the central hub in both settings, suggesting that—once detection-platform asymmetry is removed—urban and rural HR-HPV co-infection ecologies are quantitatively similar. When the comparison was extended to each cohort’s full panel (Supplementary Figure S1), the urban mean degree rose to 17.3, but this increase is largely attributable to the 10 additional low-risk and probable-high-risk genotypes detectable only by the urban Sansure 25-type panel rather than to a substantive urban–rural difference in co-infection ecology. The hub centrality of HPV52 in both settings is consistent with its dominant prevalence in this region and may reflect shared transmission routes or complementary immune-evasion strategies that facilitate its co-occurrence with multiple other high-risk types.

## Recommendations

### Vaccination strategy optimization

Our findings support reconsideration of HPV vaccination strategies in China. Whereas current bivalent and quadrivalent vaccines target only HPV16/18, the nine-valent vaccine (Gardasil 9) additionally targets HPV31/33/45/52/58—including HPV52 and HPV58, which dominate the high-risk genotype landscape in our cohort (Table 3). The substantial expansion of genotype coverage achievable with nine-valent vaccination in populations where HPV52 and HPV58 are highly prevalent is consistent with population-level impact studies in Asian settings [23, 51]. Whether this translates into a proportionate reduction in cervical cancer incidence in Northwest China will depend on local cost-effectiveness modelling, which is identified here as a research priority but is beyond the scope of the present study. We note, however, that our PCR-based platform identifies HPV genotypes but does not resolve viral lineages or sublineages [19]; formal demonstration that the vaccine covers the specific HPV52/58 sublineages circulating in Xi’an and Northwest China would require variant-level sequencing, which was beyond our scope. Lineage-specific cross-protection therefore remains an open question for future variant-resolved studies.

### Screening program enhancement

The extremely low rural pathological follow-up rate (11.5%; *n* = 70/609 HPV-positive rural women) represents a critical failure point in the screening-to-treatment cascade within the rural sub-cohort. This is substantially worse than the WHO-recommended minimum of 70% follow-up for HPV-positive women [24] and far below rates achieved in high-income countries. Matched urban follow-up cascade data were not available for the present analysis, which precludes formal urban–rural comparison of cascade-of-care indicators; we therefore confine cascade-of-care interpretations to the rural sub-cohort and identify matched-cohort urban–rural cascade evaluation as an immediate priority for follow-up studies.

Addressing this requires multipronged interventions: automated recall systems using SMS or mobile apps, integration of HPV screening with follow-up scheduling, establishment of regional colposcopy centers to reduce geographic barriers, and patient education emphasizing that positive HPV results require but do not guarantee cancer development. Comparative evaluation of community-based versus facility-based follow-up models is identified as a priority for subsequent prospective studies.

### Pathology outcome distribution in the rural follow-up subset (transparency disclosure)

Following the formal withdrawal of the previously circulated 3.8-fold higher CIN2+ risk associated with HPV16/18 detection (see Limitations and Supplementary Table S2), this subsection discloses the corrected pathology distribution in the rural follow-up subset for transparency only; the data presented below are descriptive observations from the rural sub-cohort (matched urban follow-up cascade data were not available) and are explicitly not intended as risk estimates. They are derived from a small confirmed-pathology subset (*n* = 70 cases, representing 11.5% pathological follow-up of *n* = 609 HPV-positive rural women); future longitudinal studies with active recall procedures and matched urban–rural follow-up will be required to validate any genotype-specific estimates before they are used for individual-level clinical decision-making. Among the *n* = 70 followed-up rural HPV-positive women, the pathological severity distribution was: Normal 56 (80.0%), ASC-US 8 (11.4%), LSIL/CIN1 1 (1.4%), HSIL/CIN2-3 2 (2.9%), Cancer 0, and non-cervical or unknown 3 (4.3%) (Supplementary Table S2). Stratified by HPV16/18 status, high-grade lesions (HSIL/CIN2-3 or Cancer) occurred in 1/21 (4.8%) of HPV16/18-positive women and 1/49 (2.0%) of HPV16/18-negative women; Fisher’s exact test for this comparison was not statistically significant (two-sided *p* = 0.513; effect-size estimation withheld per Supplementary Table S2 Note ^2^). Following this internal data audit and re-analysis, we have therefore withdrawn the previously reported point estimates for HPV16/18-specific CIN2+ risk and for genotype-by-multiplicity and genotype-by-age progression effects: the corrected *n* = 70 rural sub-cohort analysis does not support a statistically significant genotype-specific high-grade lesion effect, and any such estimates from a subset of this size and selection structure would be unreliable. We retain the conceptual recommendation—that future risk-stratification frameworks should integrate genotype, infection multiplicity, and age—but we now present this as a hypothesis to be tested in larger, prospectively followed cohorts with matched urban–rural pathology cascade, rather than as a finding supported by the present data. Selection-bias diagnostics for this subset are reported in Supplementary Table S1 (see also Limitations).

Pending such evidence, individual-level screening management for HPV-positive women in this population should follow established national guidelines [35, 40] rather than be modified on the basis of the small descriptive subset reported here. Whether risk-stratified screening intervals incorporating genotype, infection multiplicity, and age can safely refine these guidelines in Chinese populations remains an open question to be addressed by adequately powered prospective studies with matched urban–rural pathology cascade.

### Temporal trends and COVID-19 Impact

The increasing HPV prevalence from 2023 to 2024 (12.2% to 14.8%, *p* = 0.011) requires careful interpretation. Given that HPV persistence operates on a timescale of years and individual infection trajectories cannot be tracked in a cross-sectional design, the 36-month study period does not capture the full temporal dynamics of infection clearance or progression. Rather, population-level temporal trends in this study reflect changes in screening coverage, healthcare-seeking behaviour, and case ascertainment—not individual infection incidence. In the context of COVID-19 pandemic recovery, studies from multiple countries reported 20–40% reductions in cervical screening during pandemic lockdowns [52], potentially leading to accumulation of undetected infections. Our observed increase may therefore represent “catch-up” screening of previously undetected cases rather than a true increase in underlying incidence. To formally evaluate this catch-up screening hypothesis, we reconstructed month-resolved screening volumes and HPV positivity for both populations across the entire 36-month observation window (Fig. 5; Supplementary Table S4). Urban screening volume peaked at *n* = 1,052 in March 2023—the highest single-month value of the series—consistent with the release of pandemic-suppressed demand following the December 2022 lifting of national pandemic-control measures. Rural screening showed two large batched waves in July 2024 (*n* = 632) and April 2025 (*n* = 607), interpretable as scheduled regional catch-up campaigns rather than steady-state utilisation. Mann–Kendall trend testing identified a statistically significant monotonic increase in HR-HPV positivity in both populations across the study period (urban Z = +2.74, *p* = 0.006; rural Z = +2.10, *p* = 0.036), and a parallel significant increase in any-HPV positivity in the urban population (Z = +2.78, *p* = 0.005); the rural any-HPV trend approached but did not reach significance (Z = +1.77, *p* = 0.077). The 2023 surge likely reabsorbed previously deferred lower-prevalence attendees, inflating the early-period denominator; as the attendee mix normalised, the higher 2024 prevalence (14.8%) is more consistent with a combination of catch-up screening of higher-risk individuals and a genuine secular increase in HR-HPV detection than with a measurement artefact alone. Wilson 95% confidence intervals and a Low_N flag for months with *N* < 30 are reported in Supplementary Table S4; in Fig. 5, months with *N* < 30 are visually de-emphasised (hollow markers; marker area proportional to sqrt(N)) so that small-sample monthly excursions—particularly in the rural cohort during late 2025—are not over-interpreted. Additionally, COVID-19-related behavioural changes may have influenced HPV transmission dynamics. Longer-term surveillance with formally validated denominator-aware monitoring (e.g., month-resolved screening-volume linkage to administrative records) remains essential to fully distinguish pandemic-specific ascertainment effects from genuine epidemiological trends; this validation is identified as a priority for follow-up studies.

## Limitations

This study has several important limitations that warrant consideration. First, the cross-sectional design precludes causal inference regarding infection dynamics and progression risk. We cannot determine whether observed HPV infections represent incident or persistent cases, limiting our ability to distinguish true high-risk populations from those with transient infections likely to clear spontaneously. Future longitudinal studies will be required to characterise infection persistence and progression in this population.

Second, the pathological follow-up data available in this cross-sectional dataset are limited to the rural sub-cohort, in which the follow-up rate was 11.5% (*n* = 70/609 HPV-positive rural women), substantially below the World Health Organization-recommended minimum of 70% follow-up for HPV-positive women within organised screening programmes [24]; matched urban follow-up cascade data were not available, which precludes any urban–rural comparison of cascade-of-care indicators or genotype-specific progression risk and confines all cascade-of-care interpretations to the rural sub-cohort. This shortfall is a major limitation of the present analysis. To document the internal validity of the limited descriptive use of the rural followed-up subset, we performed a selection bias analysis (Supplementary Table S1) comparing the *n* = 70 followed-up rural women with the *n* = 539 not-followed-up rural women across baseline covariates: no statistically significant differences were observed on age (50.1 ± 9.4 vs. 48.7 ± 10.6 years; Welch’s *t* = 1.221, *p* = 0.225), age group distribution (χ^2^ = 6.800, *p* = 0.147), infection multiplicity (χ^2^ = 0.338, *p* = 0.561), or HPV16/18 status (χ^2^ = 1.769, *p* = 0.184), supporting that the followed-up subset is representative of the broader rural HPV-positive cohort on observed covariates even though absolute follow-up rate is well below the WHO benchmark. Following internal data audit, we have additionally withdrawn the previously reported HPV16/18-specific CIN2+ risk estimate (3.8-fold), as the corrected *n* = 70 analysis did not support a statistically significant genotype-specific high-grade lesion effect (Fisher’s exact *p* = 0.513; Supplementary Table S2); we now present pathology outcomes descriptively only, with all cascade-of-care and genotype-specific interpretations explicitly framed as preliminary, rural-only descriptive observations. We have positioned the present work as a population-based epidemiological survey of HPV prevalence, genotype distribution, and screening engagement, rather than as a complete outcome evaluation of a screening programme. Future longitudinal studies with active recall procedures and matched urban–rural pathology follow-up will be required to validate any genotype-specific risk estimates and to assess whether the observed cascade-of-care gap can be narrowed toward the WHO benchmark.

Third, the observational nature and potential for unmeasured confounding limit our mechanistic interpretations. We lacked data on important behavioral factors including number of lifetime sexual partners, age at sexual debut, smoking status, and oral contraceptive use, all of which influence HPV infection and persistence risk. Socioeconomic status was categorized only by urban/rural residence, masking potentially important within-group heterogeneity. Future studies incorporating detailed behavioral and socioeconomic data would strengthen causal inference.

Fourth, the urban and rural cohorts differed in both size and age composition: the urban sample was substantially larger (*n* = 11,449 vs *n* = 4,916) and younger on average (mean age 42.8 vs. 47.0 years). Although age-stratified analyses and multivariable adjustments were applied, residual imbalance in age structure and statistical power between settings may still influence direct urban–rural comparisons of crude prevalence and genotype distribution, and limits the precision of stratum-specific estimates in older age bands of the rural cohort. Stratified estimates and subgroup analyses should therefore be interpreted as describing the populations actually screened in each setting under existing programmatic conditions, rather than as fully matched epidemiological comparisons. Future studies with balanced urban–rural recruitment and harmonised age structures will be required to refine these comparative estimates.

Fifth, because this study used retrospectively extracted hospital screening records, individual-level information on potentially relevant clinical exclusion conditions—including previous hysterectomy, current pregnancy, menstruation at the time of sampling, prior cervical cancer, and recent cervical procedures within 3 months—was not consistently available in the source datasets and could not be applied at the record level. The analytical cohort was therefore restricted on the basis of female sex, presence of a numerically valid age within 15–100 years, and uniqueness of testing record. Records with missing or non-numeric age were excluded jointly with out-of-range ages via numeric range filtering on the age field. Although the resulting population is broadly representative of women presenting for HPV testing under routine programmatic conditions in the participating facilities, residual heterogeneity introduced by the absence of these record-level clinical filters cannot be formally excluded; given the large sample size, this is unlikely to materially affect overall prevalence estimates but should be borne in mind when interpreting subgroup comparisons.

Despite these limitations, and acknowledging that the present work is best characterised as a population-based epidemiological survey rather than a complete evaluation of a fully implemented screening programme, this study provides a population-based characterisation of urban–rural disparities in HPV prevalence, genotype distribution, and screening engagement in a large Chinese setting, with implications for nine-valent vaccination policy and for prioritising follow-up infrastructure. The large sample size, comprehensive 25-type genotyping, population-based recruitment, and methodologically standardised urban–rural comparison support the validity of our principal epidemiological findings regarding HPV prevalence, genotype distribution, and age-specific patterns. Confirmatory longitudinal evidence on cascade-of-care outcomes will be required to complement the cross-sectional findings reported here.

## Electronic supplementary material

Below is the link to the electronic supplementary material.

Supplementary material 1

## Data Availability

The datasets used and analyzed during the current study are not publicly available due to restrictions related to patient privacy and institutional regulations but are available from the corresponding author on reasonable request.
