# Supplementary material for: Urban–rural disparities in HPV prevalence and genotype distribution: a large-scale cervical cancer screening study in Xi’an, China
Source: BMC Infect Dis. 2026 May 28;26:1308. doi: 10.1186/s12879-026-13647-2 (PMC13361063; doi:10.1186/s12879-026-13647-2)
Supplement: Supplementary file 1 — Supplementary material 1 [file 12879_2026_13647_MOESM1_ESM.docx]

**Additional File 1: Supplementary Materials**

Urban–Rural Disparities in HPV Prevalence and Genotype Distribution: A Large-Scale Cervical Cancer Screening Study in Xi'an, China

Wu et al., BMC Infectious Diseases, 2026

**Contents**

**• Supplementary Table S1.** Selection-bias diagnostic: comparison of baseline characteristics between rural HPV-positive women with and without subsequent pathological follow-up (n = 609).

**• Supplementary Table S2.** Histopathological outcomes stratified by HPV16/18 infection status in the rural follow-up subset (n = 70).

**• Supplementary Figure S1.** Full-panel HPV co-infection network architecture: urban (25-type Sansure panel) vs rural (15-type panel).

**• Supplementary Table S3.** Full-panel HPV co-infection network statistics (companion data to Supplementary Figure S1).

**• Supplementary Table S4.** Monthly screening volume and HPV positivity, urban vs rural, January 2023 – December 2025 (n = 16,365 women); companion data table for Figure 8.

**Supplementary Table S1. Selection-bias diagnostic in rural HPV-positive women (n = 609).**

| **Variable** | **Followed up (n = 70)** | **Not followed up (n = 539)** | **Test statistic** | **P value** | **Statistical test** |
| --- | --- | --- | --- | --- | --- |
|  | **n (%)** | **n (%)** |  |  |  |
| Total, n | 70 | 539 |  |  |  |
| Age, mean ± SD (years) | 50.1 ± 9.4 | 48.7 ± 10.6 | t = 1.221 | 0.225 | Welch's t-test |
| Age, median [IQR] (years) | 53 [43–57] | 50 [40–57] | U = 20,409 | 0.265 | Mann–Whitney U |
| Age group, n (%)¹ |  |  | χ² = 6.800 | 0.147 | Pearson's χ² |
| <30 | 1 (1.4) | 19 (3.5) |  |  |  |
| 30–39 | 10 (14.3) | 108 (20.0) |  |  |  |
| 40–49 | 15 (21.4) | 133 (24.7) |  |  |  |
| 50–59 | 35 (50.0) | 187 (34.7) |  |  |  |
| ≥60 | 9 (12.9) | 92 (17.1) |  |  |  |
| Infection multiplicity, n (%) |  |  | χ² = 0.338 | 0.561 | Pearson's χ² |
| Single-type infection | 49 (70.0) | 395 (73.3) |  |  |  |
| Multiple-type infection | 21 (30.0) | 144 (26.7) |  |  |  |
| HPV16/18 status, n (%) |  |  | χ² = 1.769 | 0.184 | Pearson's χ² |
| HPV16/18 negative | 49 (70.0) | 416 (77.2) |  |  |  |
| HPV16/18 positive | 21 (30.0) | 123 (22.8) |  |  |  |

**Notes.**

Data are presented as n (%) for categorical variables and as mean ± SD or median [IQR] for continuous variables.

Welch's two-sample t-test was used for unequal-variance comparison of means; Mann–Whitney U test for distributional comparison of medians; Pearson's χ² test (without continuity correction) for k × 2 contingency tables, with Fisher's exact test substituted when any expected cell count was < 5. All tests were two-sided.

The pathological follow-up rate among rural HPV-positive women was 70/609 (11.5%), well below the WHO-recommended minimum of 70% for completion of the screening-to-treatment cascade. None of the measured baseline characteristics differed significantly between the followed-up and non-followed-up groups (all P > 0.10), supporting representativeness of the followed-up subset on the observed covariates and informing the interpretation of Supplementary Table S2.

¹ Age-group percentages are computed against the followed-up column total (n = 70) and the not-followed-up column total (n = 539); the 5 × 2 cross-tabulation was tested using Pearson's χ² without continuity correction. The rural analytic cohort (n = 4,915 / 609 HPV-positive) corresponds to the main manuscript (Table 1; n = 4,916 / 609) after applying the pre-specified eligibility filter (age ≥ 18 years and complete demographic data); 9 HPV-positive rural records with missing age were excluded under "incomplete demographic data" per the Methods section.

HPV positivity was defined as detection of any high-risk HPV genotype; multiplicity was defined as detection of two or more genotypes; HPV16/18 status was defined as detection of HPV16 and/or HPV18.

Matched urban follow-up cascade data were not available for the present analysis; consequently, no urban–rural comparison of cascade-of-care outcomes could be performed.

Data source: institutional cervical cancer screening database, rural sub-cohort, January 2023 – December 2025.

Abbreviations: HPV, human papillomavirus; HR-HPV, high-risk HPV; SD, standard deviation; IQR, interquartile range; WHO, World Health Organization.

**Supplementary Table S2. Pathology outcomes stratified by HPV16/18 status (n = 70).**

| **Pathology category** | **HPV16/18 negative (n = 49)** | **HPV16/18 positive (n = 21)** | **Total (n = 70)** | **Test statistic¹** | **P value¹** |
| --- | --- | --- | --- | --- | --- |
|  | **n (%)** | **n (%)** | **n (%)** |  |  |
| Normal cytology | 43 (87.8) | 13 (61.9) | 56 (80.0) |  |  |
| *Abnormal cytology* |  |  |  |  |  |
| ASC-US | 4 (8.2) | 4 (19.0) | 8 (11.4) |  |  |
| ASC-H | 0 (0.0) | 0 (0.0) | 0 (0.0) |  |  |
| LSIL/CIN1 | 0 (0.0) | 1 (4.8) | 1 (1.4) |  |  |
| HSIL/CIN2-3 | 1 (2.0) | 1 (4.8) | 2 (2.9) |  |  |
| Cervical cancer | 0 (0.0) | 0 (0.0) | 0 (0.0) |  |  |
| High-grade lesion² | 1 (2.0) | 1 (4.8) | 2 (2.9) | — | 0.513 |
| Non-cervical lesion | 1 (2.0) | 1 (4.8) | 2 (2.9) |  |  |
| Unknown / Other | 0 (0.0) | 1 (4.8) | 1 (1.4) |  |  |

**Notes.**

Data are presented as n (%); column percentages are computed within each HPV16/18 stratum or over the total follow-up subset.

¹ Statistical comparison was pre-specified for the high-grade lesion outcome only and performed with Fisher's exact test (two-sided), as per-stratum cell counts in the other categories were too small for stable inference. Fisher's exact test does not yield a parametric test statistic; the two-sided P value is therefore the only inferential summary reported, and effect-size estimation was withheld (Note ²) to avoid unstable interval estimates given the small follow-up sample. The high-grade lesion outcome was defined a priori as HSIL/CIN2-3 or invasive cervical cancer.

² Effect-size confidence intervals were intentionally not estimated for the categorical pathology outcome to avoid unstable interval estimates given the small follow-up sample size.

Diagnoses were prioritised using the adjudicated revised diagnosis (integrating cytology and histology) over the original standard diagnosis where both were available.

The follow-up subset comprised 70 of 609 rural HPV-positive women (11.5%); the corresponding selection-bias diagnostics are reported in Supplementary Table S1.

Data-audit disclosure: a previously circulated preliminary estimate of an approximately 3.8-fold higher CIN2+ risk associated with HPV16/18 detection (P < 0.001), based on an outdated subset (n = 87), has been formally withdrawn following internal data audit and is superseded by the present, audited analysis.

Matched urban follow-up cascade data were not available for the present study, which precludes any urban–rural comparison of cascade-of-care outcomes or genotype-specific progression risk. Future longitudinal studies with active recall procedures and matched urban–rural pathology follow-up will be required to validate any genotype-specific risk estimates in this population.

Abbreviations: HPV, human papillomavirus; ASC-US, atypical squamous cells of undetermined significance; ASC-H, atypical squamous cells, cannot exclude high-grade squamous intraepithelial lesion; LSIL, low-grade squamous intraepithelial lesion; HSIL, high-grade squamous intraepithelial lesion; CIN, cervical intraepithelial neoplasia.

**Supplementary Figure S1. Full-panel HPV co-infection network architecture: urban (25-type Sansure panel) vs rural (15-type panel)**

**

**

**Notes.**

HPV genotype co-infection networks under each cohort's complementary detection panel: urban (25-type Sansure panel; n = 1,780 HPV-positive women) and rural (15-type HR-HPV-only panel; n = 609 HPV-positive women). Node size proportional to detection frequency within each cohort; node colour indicates the IARC carcinogenicity classification [50,51]: red, IARC Group 1 "carcinogenic to humans" (HPV16, 18, 31, 33, 35, 39, 45, 51, 52, 56, 58, 59) plus Group 2A "probably carcinogenic" (HPV68); orange, IARC Group 2B "possibly carcinogenic" (HPV53 and HPV66 detectable in both panels; HPV26, 70, 73, 82 detectable in the urban 25-type panel only); green, low-risk / non-carcinogenic genotypes (HPV6, 11, 42, 43, 81, 83). Edge width proportional to co-occurrence frequency; numerical edge labels denote co-occurrence count for pairs with at least 5 shared cases. Network statistics (corrected to the standard 2 × edges / nodes formula): urban 23 nodes, 199 edges, mean degree 17.3; rural 15 nodes, 94 edges, mean degree 12.5; ratio 1.38. Caveat: the apparent higher urban density observed in the full-panel view is largely attributable to the 10 additional non-platform-shared genotypes detectable only by the urban Sansure 25-type panel (4 IARC Group 2B: HPV26, 70, 73, 82; and 6 low-risk: HPV6, 11, 42, 43, 81, 83) and is therefore not directly density-comparable; the platform-comparable 15-vs-15 network reported in main-text Figure 6 (with the same node set in both cohorts) is taken as the primary analytic comparison. Companion data are reported in Supplementary Table S3.

**Supplementary Table S3. Full-panel HPV co-infection network statistics (companion data to Supplementary Figure S1).**

| **A. HPV Genotype Frequency Comparison** | | | | |
| --- | --- | --- | --- | --- |
| **HPV Type** | **Urban (n)** | **Urban (%)** | **Rural (n)** | **Rural (%)** |
| HPV52 | 352 | 19.8 | 158 | 25.9 |
| HPV58 | 277 | 15.6 | 80 | 13.1 |
| HPV16 | 271 | 15.2 | 119 | 19.5 |
| HPV53 | 232 | 13.0 | 93 | 15.3 |
| HPV42 | 128 | 7.2 | 0 | 0.0 |
| HPV68 | 122 | 6.9 | 51 | 8.4 |
| HPV51 | 120 | 6.7 | 64 | 10.5 |
| HPV56 | 107 | 6.0 | 57 | 9.4 |
| HPV81 | 94 | 5.3 | 0 | 0.0 |
| HPV66 | 92 | 5.2 | 38 | 6.2 |
| HPV18 | 80 | 4.5 | 28 | 4.6 |
| HPV31 | 80 | 4.5 | 30 | 4.9 |
| HPV39 | 76 | 4.3 | 52 | 8.5 |
| HPV59 | 67 | 3.8 | 26 | 4.3 |
| HPV33 | 64 | 3.6 | 49 | 8.0 |
| HPV6 | 63 | 3.5 | 0 | 0.0 |
| HPV35 | 62 | 3.5 | 15 | 2.5 |
| HPV11 | 30 | 1.7 | 0 | 0.0 |
| HPV82 | 24 | 1.3 | 0 | 0.0 |
| HPV70 | 23 | 1.3 | 0 | 0.0 |
| **B. Network Statistics Comparison** | | | | |
| **Metric** | **Urban** | **—** | **Rural** | **—** |
| Total Positive Cases | 1,780 | — | 609 | — |
| Network Nodes | 23 | — | 15 | — |
| Network Edges | 199 | — | 94 | — |
| **C. Top Co-Infection Pairs** | | | | |
| **Rank** | **Urban Pair** | **Count** | **Rural Pair** | **Count** |
| 1 | HPV52+HPV53 | 32 | HPV52+HPV53 | 16 |
| 2 | HPV58+HPV53 | 25 | HPV51+HPV52 | 14 |
| 3 | HPV16+HPV52 | 23 | HPV52+HPV58 | 14 |
| 4 | HPV52+HPV58 | 21 | HPV52+HPV56 | 12 |
| 5 | HPV58+HPV42 | 21 | HPV16+HPV52 | 10 |
| 6 | HPV52+HPV66 | 18 | HPV16+HPV53 | 10 |
| 7 | HPV16+HPV53 | 15 | HPV52+HPV68 | 10 |
| 8 | HPV39+HPV68 | 15 | HPV33+HPV53 | 9 |
| 9 | HPV52+HPV68 | 15 | HPV39+HPV51 | 9 |
| 10 | HPV68+HPV53 | 15 | HPV39+HPV52 | 9 |

**Notes.**

Companion data table for Supplementary Figure S1. Urban: 25-type Sansure panel (n = 1,780 HPV-positive women); Rural: 15-type HR-HPV-only panel (n = 609 HPV-positive women). Section A: HPV genotype frequency among HPV-positive cases within each cohort, restricted to genotypes detected in the respective panel. Section B: network-level summary (total HPV-positive cases, network nodes, network edges). Section C: top 10 co-infection pairs ranked by co-occurrence count. Mean degree (2 × edges / nodes): 17.3 urban, 12.5 rural (ratio 1.38). The platform-comparable primary network analysis (15 platform-shared high-risk genotypes; mean degree 12.7 vs 12.5; ratio 1.02) is reported in main-text Table 5 and Figure 6.

**Supplementary Table S4. Monthly screening volume and HPV positivity by setting, January 2023 – December 2025.**

| **Year–Month** | **Setting** | **Screened (N)** | **Any-HPV+ n** | **Any-HPV+ % (95% CI)** | | **HR-HPV+ n** | **HR-HPV+ % (95% CI)** | **Low N¹** |
| --- | --- | --- | --- | --- | --- | --- | --- | --- |
| **Urban (n = 11,449 women)** | | | | | | | | |
| 2023-01 | Urban | 67 | 12 | 17.9 (10.6–28.7) | | 10 | 14.9 (8.3–25.3) |  |
| 2023-02 | Urban | 449 | 45 | 10.0 (7.6–13.1) | | 32 | 7.1 (5.1–9.9) |  |
| 2023-03 | Urban | 1,052 | 93 | 8.8 (7.3–10.7) | | 81 | 7.7 (6.2–9.5) |  |
| 2023-04 | Urban | 641 | 74 | 11.5 (9.3–14.3) | | 61 | 9.5 (7.5–12.0) |  |
| 2023-05 | Urban | 355 | 38 | 10.7 (7.9–14.4) | | 29 | 8.2 (5.7–11.5) |  |
| 2023-06 | Urban | 214 | 34 | 15.9 (11.6–21.4) | | 29 | 13.6 (9.6–18.8) |  |
| 2023-07 | Urban | 175 | 36 | 20.6 (15.2–27.2) | | 31 | 17.7 (12.8–24.0) |  |
| 2023-08 | Urban | 287 | 41 | 14.3 (10.7–18.8) | | 33 | 11.5 (8.3–15.7) |  |
| 2023-09 | Urban | 292 | 38 | 13.0 (9.6–17.4) | | 32 | 11.0 (7.9–15.1) |  |
| 2023-10 | Urban | 241 | 44 | 18.3 (13.9–23.6) | | 28 | 11.6 (8.2–16.3) |  |
| 2023-11 | Urban | 469 | 55 | 11.7 (9.1–15.0) | | 40 | 8.5 (6.3–11.4) |  |
| 2023-12 | Urban | 232 | 36 | 15.5 (11.4–20.7) | | 26 | 11.2 (7.8–15.9) |  |
| **Urban total 2023** |  | **4,474** | **546** | **12.2 (11.3–13.2)** | | **432** | **9.7 (8.8–10.6)** |  |
| 2024-01 | Urban | 202 | 36 | 17.8 (13.2–23.7) | | 27 | 13.4 (9.4–18.7) |  |
| 2024-02 | Urban | 132 | 39 | 29.5 (22.4–37.8) | | 28 | 21.2 (15.1–28.9) |  |
| 2024-03 | Urban | 367 | 60 | 16.3 (12.9–20.5) | | 40 | 10.9 (8.1–14.5) |  |
| 2024-04 | Urban | 384 | 62 | 16.1 (12.8–20.2) | | 48 | 12.5 (9.6–16.2) |  |
| 2024-05 | Urban | 301 | 64 | 21.3 (17.0–26.2) | | 47 | 15.6 (12.0–20.1) |  |
| 2024-06 | Urban | 317 | 47 | 14.8 (11.3–19.2) | | 35 | 11.0 (8.0–15.0) |  |
| 2024-07 | Urban | 466 | 63 | 13.5 (10.7–16.9) | | 54 | 11.6 (9.0–14.8) |  |
| 2024-08 | Urban | 325 | 40 | 12.3 (9.2–16.3) | | 23 | 7.1 (4.8–10.4) |  |
| 2024-09 | Urban | 219 | 43 | 19.6 (14.9–25.4) | | 35 | 16.0 (11.7–21.4) |  |
| 2024-10 | Urban | 213 | 44 | 20.7 (15.8–26.6) | | 35 | 16.4 (12.1–22.0) |  |
| 2024-11 | Urban | 218 | 36 | 16.5 (12.2–22.0) | | 29 | 13.3 (9.4–18.5) |  |
| 2024-12 | Urban | 220 | 55 | 25.0 (19.7–31.1) | | 45 | 20.5 (15.7–26.3) |  |
| **Urban total 2024** |  | **3,364** | **589** | **17.5 (16.3–18.8)** | | **446** | **13.3 (12.2–14.4)** |  |
| 2025-01 | Urban | 155 | 34 | 21.9 (16.1–29.1) | | 28 | 18.1 (12.8–24.9) |  |
| 2025-02 | Urban | 183 | 37 | 20.2 (15.0–26.6) | | 22 | 12.0 (8.1–17.5) |  |
| 2025-03 | Urban | 278 | 55 | 19.8 (15.5–24.9) | | 49 | 17.6 (13.6–22.5) |  |
| 2025-04 | Urban | 292 | 59 | 20.2 (16.0–25.2) | | 39 | 13.4 (9.9–17.7) |  |
| 2025-05 | Urban | 201 | 43 | 21.4 (16.3–27.6) | | 36 | 17.9 (13.2–23.8) |  |
| 2025-06 | Urban | 183 | 45 | 24.6 (18.9–31.3) | | 36 | 19.7 (14.6–26.0) |  |
| 2025-07 | Urban | 338 | 57 | | 16.9 (13.2–21.2) | 46 | 13.6 (10.4–17.7) |  |
| 2025-08 | Urban | 257 | 47 | 18.3 (14.0–23.5) | | 36 | 14.0 (10.3–18.8) |  |
| 2025-09 | Urban | 378 | 62 | 16.4 (13.0–20.5) | | 46 | 12.2 (9.2–15.9) |  |
| 2025-10 | Urban | 210 | 49 | 23.3 (18.1–29.5) | | 40 | 19.0 (14.3–24.9) |  |
| 2025-11 | Urban | 315 | 52 | 16.5 (12.8–21.0) | | 47 | 14.9 (11.4–19.3) |  |
| 2025-12 | Urban | 821 | 105 | 12.8 (10.7–15.2) | | 88 | 10.7 (8.8–13.0) |  |
| **Urban total 2025** |  | **3,611** | **645** | **17.9 (16.6–19.1)** | | **513** | **14.2 (13.1–15.4)** |  |
| **Urban total 2023–2025** |  | **11,449** | **1,780** | **15.5 (14.9–16.2)** | | **1,391** | **12.1 (11.6–12.8)** |  |
| **Rural (n = 4,916 women)** | | | | | | | | |
| 2023-01 | Rural | 13 | 0 | 0.0 (0.0–22.8) | | 0 | 0.0 (0.0–22.8) | Yes |
| 2023-02 | Rural | 84 | 7 | 8.3 (4.1–16.2) | | 6 | 7.1 (3.3–14.7) |  |
| 2023-03 | Rural | 224 | 29 | 12.9 (9.2–18.0) | | 25 | 11.2 (7.7–16.0) |  |
| 2023-04 | Rural | 142 | 14 | 9.9 (6.0–15.9) | | 13 | 9.2 (5.4–15.0) |  |
| 2023-05 | Rural | 163 | 12 | 7.4 (4.3–12.4) | | 10 | 6.1 (3.4–10.9) |  |
| 2023-06 | Rural | 202 | 23 | 11.4 (7.7–16.5) | | 20 | 9.9 (6.5–14.8) |  |
| 2023-07 | Rural | 165 | 21 | 12.7 (8.5–18.7) | | 19 | 11.5 (7.5–17.3) |  |
| 2023-08 | Rural | 28 | 8 | 28.6 (15.3–47.1) | | 7 | 25.0 (12.7–43.4) | Yes |
| 2023-09 | Rural | 21 | 4 | 19.0 (7.7–40.0) | | 3 | 14.3 (5.0–34.6) | Yes |
| 2023-10 | Rural | 37 | 7 | 18.9 (9.5–34.2) | | 6 | 16.2 (7.7–31.1) |  |
| 2023-11 | Rural | 23 | 7 | 30.4 (15.6–50.9) | | 6 | 26.1 (12.5–46.5) | Yes |
| 2023-12 | Rural | 26 | 3 | 11.5 (4.0–29.0) | | 3 | 11.5 (4.0–29.0) | Yes |
| **Rural total 2023** |  | **1,128** | **135** | **12.0 (10.2–14.0)** | | **118** | **10.5 (8.8–12.4)** |  |
| 2024-01 | Rural | 57 | 8 | 14.0 (7.3–25.3) | | 6 | 10.5 (4.9–21.1) |  |
| 2024-02 | Rural | 31 | 3 | 9.7 (3.3–24.9) | | 2 | 6.5 (1.8–20.7) |  |
| 2024-03 | Rural | 70 | 10 | 14.3 (7.9–24.3) | | 10 | 14.3 (7.9–24.3) |  |
| 2024-04 | Rural | 85 | 9 | 10.6 (5.7–18.9) | | 9 | 10.6 (5.7–18.9) |  |
| 2024-05 | Rural | 101 | 11 | 10.9 (6.2–18.5) | | 9 | 8.9 (4.8–16.1) |  |
| 2024-06 | Rural | 345 | 37 | 10.7 (7.9–14.4) | | 32 | 9.3 (6.6–12.8) |  |
| 2024-07 | Rural | 632 | 68 | 10.8 (8.6–13.4) | | 61 | 9.7 (7.6–12.2) |  |
| 2024-08 | Rural | 298 | 29 | 9.7 (6.9–13.6) | | 27 | 9.1 (6.3–12.9) |  |
| 2024-09 | Rural | 285 | 27 | 9.5 (6.6–13.4) | | 21 | 7.4 (4.9–11.0) |  |
| 2024-10 | Rural | 157 | 26 | 16.6 (11.6–23.2) | | 22 | 14.0 (9.4–20.3) |  |
| 2024-11 | Rural | 329 | 35 | 10.6 (7.7–14.4) | | 32 | 9.7 (7.0–13.4) |  |
| 2024-12 | Rural | 275 | 42 | 15.3 (11.5–20.0) | | 38 | 13.8 (10.2–18.4) |  |
| **Rural total 2024** |  | **2,665** | **305** | **11.4 (10.3–12.7)** | | **269** | **10.1 (9.0–11.3)** |  |
| 2025-01 | Rural | 49 | 5 | 10.2 (4.4–21.8) | | 5 | 10.2 (4.4–21.8) |  |
| 2025-02 | Rural | 61 | 17 | 27.9 (18.2–40.2) | | 14 | 23.0 (14.2–34.9) |  |
| 2025-03 | Rural | 106 | 10 | 9.4 (5.2–16.5) | | 8 | 7.5 (3.9–14.2) |  |
| 2025-04 | Rural | 607 | 80 | 13.2 (10.7–16.1) | | 75 | 12.4 (10.0–15.2) |  |
| 2025-05 | Rural | 98 | 14 | 14.3 (8.7–22.6) | | 13 | 13.3 (7.9–21.4) |  |
| 2025-06 | Rural | 56 | 10 | 17.9 (10.0–29.8) | | 9 | 16.1 (8.7–27.8) |  |
| 2025-07 | Rural | 27 | 3 | 11.1 (3.9–28.1) | | 3 | 11.1 (3.9–28.1) | Yes |
| 2025-08 | Rural | 35 | 5 | 14.3 (6.3–29.4) | | 5 | 14.3 (6.3–29.4) |  |
| 2025-09 | Rural | 22 | 6 | 27.3 (13.2–48.2) | | 6 | 27.3 (13.2–48.2) | Yes |
| 2025-10 | Rural | 19 | 7 | 36.8 (19.1–59.0) | | 7 | 36.8 (19.1–59.0) | Yes |
| 2025-11 | Rural | 23 | 10 | 43.5 (25.6–63.2) | | 9 | 39.1 (22.2–59.2) | Yes |
| 2025-12 | Rural | 20 | 2 | 10.0 (2.8–30.1) | | 1 | 5.0 (0.9–23.6) | Yes |
| **Rural total 2025** |  | **1,123** | **169** | **15.0 (13.1–17.3)** | | **155** | **13.8 (11.9–15.9)** |  |
| **Rural total 2023–2025** |  | **4,916** | **609** | **12.4 (11.5–13.3)** | | **542** | **11.0 (10.2–11.9)** |  |

**Notes.**

Data are presented as monthly counts of cervical cancer screening tests performed and the corresponding number and percentage of HPV-positive results, separately for the urban and rural cohorts and aggregated by calendar year and across the full study period (January 2023 – December 2025).

Any-HPV+ refers to detection of any HPV genotype on the platform-comparable 15-genotype framework (HPV16, 18, 31, 33, 35, 39, 45, 51, 52, 53, 56, 58, 59, 66, 68); HR-HPV+ refers to detection of any of the 13 IARC Group 1/2A high-risk genotypes (HPV16, 18, 31, 33, 35, 39, 45, 51, 52, 56, 58, 59, 68) within that framework.

95% confidence intervals (CIs) for monthly and annual proportions were computed using the Wilson score method, which provides stable interval coverage near boundaries and for small denominators.

¹ Low N flag (Low N = "Yes") identifies months with fewer than 30 screened individuals (N < 30); monthly point estimates from these months are statistically unstable and should not be interpreted in isolation. Such months are visually de-emphasised in Figure 8 (hollow circular markers; marker area in Figure 8 is rendered proportional to sqrt(N)).

Mann–Kendall non-parametric tests for monotonic temporal trend were performed on the 36-month series of monthly positivity rates for each region: any-HPV positivity (urban Z = +2.78, P = 0.005; rural Z = +1.77, P = 0.077) and HR-HPV positivity (urban Z = +2.74, P = 0.006; rural Z = +2.10, P = 0.036).

Em-dashes ("—") indicate undefined values when the monthly denominator was zero.

Data source: institutional cervical cancer screening database, urban (n = 11,449 women, 11,449 testing records) and rural (n = 4,916 women, 4,916 testing records) cohorts, January 2023 – December 2025.

Abbreviations: HPV, human papillomavirus; HR-HPV, high-risk human papillomavirus; CI, confidence interval; IARC, International Agency for Research on Cancer; N, denominator (number screened); n, numerator (number of HPV-positive results).
